# A conserved SNP variation in the pre-*miR396c* flanking region in *Oryza sativa indica* landraces correlates with mature miRNA abundance

**DOI:** 10.1038/s41598-023-28836-1

**Published:** 2023-02-07

**Authors:** Deepa Jaganathan, Raja Rajakani, Dadakhalandar Doddamani, Divya Saravanan, Shalini Pulipati, Vivek Hari Sundar G, Gothandapani Sellamuthu, Shilpha Jayabalan, Kumkum Kumari, Pavithra Parthasarathy, Punitha S., Sivaprakash Ramalingam, Padubidri V. Shivaprasad, Gayatri Venkataraman

**Affiliations:** 1 Plant Molecular Biology Laboratory, Department of Biotechnology, M. S. Swaminathan Research Foundation (MSSRF), Chennai, Tamil Nadu 600113 India; 2 The Roslin Institute, University of Edinburgh, Easter Bush, Edinburgh, EH25 9RG UK; 3 Tamil Nadu Agricultural University (TNAU), Coimbatore, Tamil Nadu 641003 India; 4 National Centre for Biological Sciences, Tata Institute of Fundamental Research, GKVK Campus, Bangalore, 560065 India; 5 Excellent Team for Mitigation (ETM), Faculty of Forestry and Wood Sciences, Czech University of Life Sciences Prague, Prague, Czechia; 6 GIS and Remote Sensing Laboratory, M. S. Swaminathan Research Foundation (MSSRF), Chennai, Tamil Nadu 600113 India; 7 CSIR-IGIB, Sukhdev Vihar, Mathura Road, New Delhi, 110025 India

**Keywords:** Biotechnology, Molecular biology, Plant sciences

## Abstract

Plant precursor miRNAs (pre-miRNA) have conserved evolutionary footprints that correlate with mode of miRNA biogenesis. In plants, base to loop and loop to base modes of biogenesis have been reported. Conserved structural element(s) in pre-miRNA play a major role in turn over and abundance of mature miRNA. Pre-miR*396c* sequences and secondary structural characteristics across *Oryza* species are presented. Based on secondary structure, twelve *Oryza* pre-miR*396c* sequences are divided into three groups, with the precursor from halophytic *Oryza coarctata* forming a distinct group. The miRNA-miRNA* duplex region is completely conserved across eleven *Oryza* species as are other structural elements in the pre-miRNA, suggestive of an evolutionarily conserved base-to-loop mode of miRNA biogenesis. SNPs within *O. coarctata* mature *miR396c* sequence and miRNA* region have the potential to alter target specificity and association with the RNA-induced silencing complex. A conserved SNP variation, rs10234287911 (G/A), identified in *O*. *sativa* pre-*miR396c* sequences alters base pairing above the miRNA-miRNA* duplex. The more stable structure conferred by the ‘A_10234287911_’ allele may promote better processing vis-à-vis the structure conferred by ‘G_10234287911_’ allele. We also examine pri- and pre-*miR396c* expression in cultivated rice under heat and salinity and their correlation with *miR396c* expression.

## Introduction

MicroRNAs (miRNAs) have emerged as important post-transcriptional and translational regulators that have been implicated in playing an important roles in a wide variety of stress conditions including heat, drought, salinity, heavy metal, chilling temperature, nutrient stress and disease^1^. In plants, miRNA genes are transcribed by RNA polymerase II to produce primary miRNA (pri-miRNA), further processed to yield precursor miRNA (pre-miRNA). Pre-miRNAs have defined stem loop secondary structures that are processed by the dicing complex. Core components of the dicing complex include DICER‐LIKE1 (DCL1/RNase III), HYPONASTIC LEAVES1 (HYL1/DRB1) and SERRATE (SE) that act to yield mature miRNA/miRNA* duplexes^2^. The miRNA-miRNA* duplex is subsequently exported to the cytoplasm where they are 2′‐O‐methylated at the 3′ end by HEN1. One strand from the duplex is incorporated into ARGONAUTE1 (AGO1) to form an active RNA‐induced silencing complex (RISC) and the complementary miRNA* is degraded. DCL proteins appear to function as molecular rulers that measure and cleave small RNA duplexes at a specific length. The precision in the position of the first cut along the precursor is of utmost importance as the second cut is usually made by measuring a fixed distance from the precursor end^3^. Based on conservation of specific structural elements, both base to loop and loop to base modes of processing have been recognized to exist in plant precursor miRNAs^3,4^

More than forty miRNA gene families are associated with abiotic stress in plants. Of these, thirteen families of miRNAs are responsive to salt and drought stress^5^. A large number of salinity regulated miRNAs have been identified in switchgrass that play either a direct or indirect role in salt stress alleviation^6^. Upregulation of *miR156*, *miR158*, *miR159*, *miR165*, *miR167*, *miR168*, *miR169*, *miR171*, *miR319*, *miR393*, *miR394*, *miR396* and downregulation of *miR397*, *miR398* has been reported under salinity stress in *Arabidopsis*^7^. While some information is available about miRNAs and their genes from cultivated rice and certain wild rice species, data on genes and information on their regulation in wild rice species under salinity is limited or in certain cases, totally lacking^8–12^. In cultivated rice, *Oryza sativa*, certain members of the *miR396* gene cluster function as negative regulators under salinity^13–16^. The phylogeny and evolution of the *miR396* family has been analysed only in certain AA genomes of *Oryza* species, including *Oryza sativa*^12^. Of the eight *MIR396* genes found in *O. sativa japonica* cv. Nipponbare, two *MIR396* genes (*osa-MIR396a* and *osa-MIR396c*, the latter on the reverse strand) are clustered in a less than 10 kb genomic region on Chromosome 2.

The genus *Oryza* is represented by 27 species, broadly divisible into 11 genome types, of which 6 are diploid (*n* = 12: AA, BB, CC, EE, FF and GG) and 5 are polyploid [*n* = 24: BBCC, CCDD, HHJJ, HHKK and KKLL^17^. In this study, we report the isolation of pre-miR*396c* sequences from wild *Oryza* species that represent seven genome types and examine their structural characteristics *vis-à-vis* their sequence. Absolute conservation of the *miR396c* duplex region and also a substantial degree of conservation of certain elements of the secondary structure of pre-*miR396c* was observed. An SNP variation (A/G; rs10234287911) was identified in the pre-*miR396c* -sequence in *O. sativa* genomes^18^ and validated in a set of forty-three *O. sativa* landraces. The effect of the SNP variation on *miR396c* expression levels under salinity and elevated temperature in *O. sativa* landraces has also been examined. In addition, expression of pri-*miR396c*, pre-*miR396c* and *miR396c* target genes under salinity and heat is also reported.

## Results

**Figure 1:**
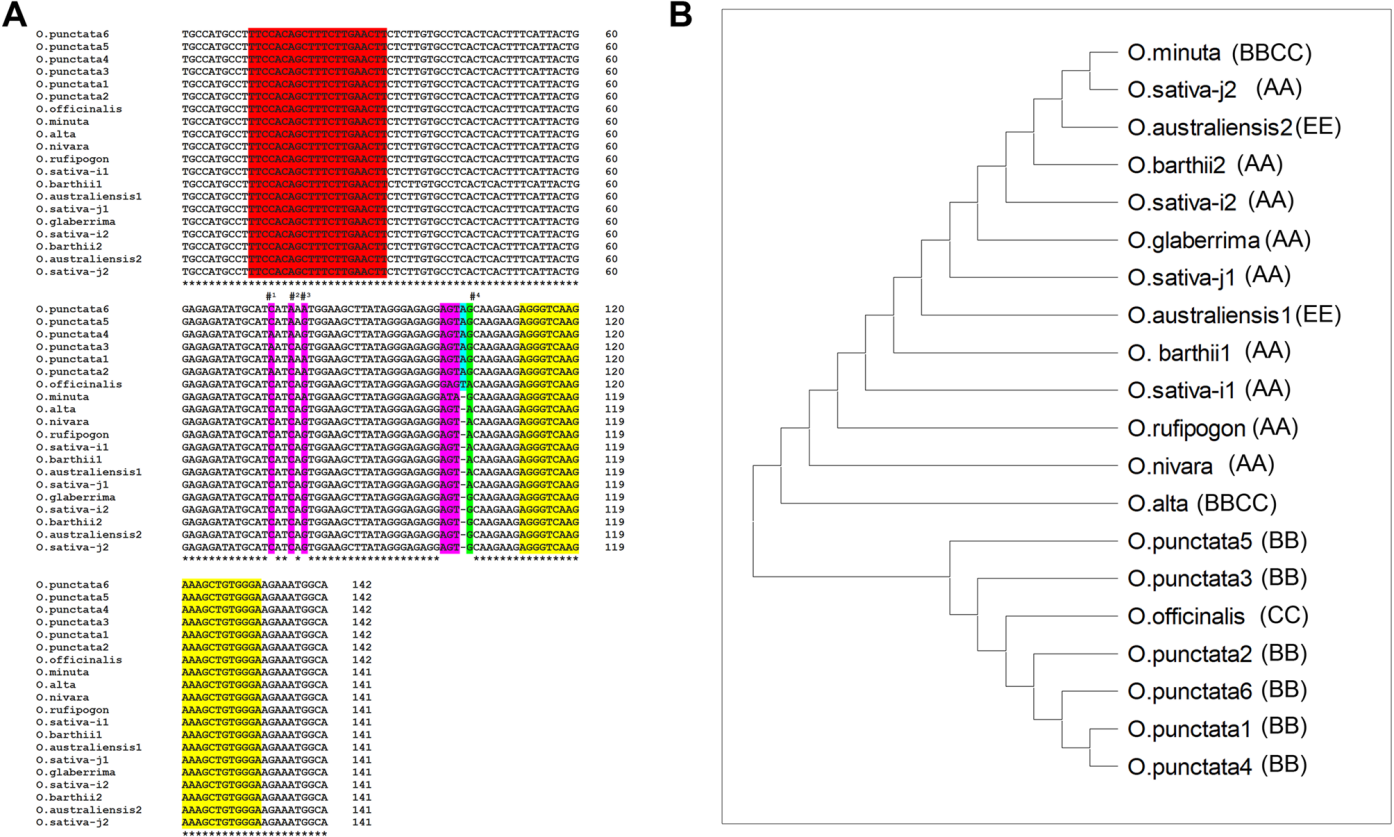
Multiple Sequence alignment (MSA) and phylogenetic analysis of pre-*miR396c* in *Oryza* species. For eleven *Oryza* species, a high degree of conservation of the pre-*miR396c* sequence was observed. (**A**)**:** Alignment of pre-*miR396c* sequences from *Oryza* species. Conservation of miRNA (Red) and miRNA* (Yellow) sequences are indicated. Seven SNPs and one indel were identified between miRNA and miRNA* regions and bases are highlighted in pink or green. Of these, residues marked #^1^-#^3^ were heterozygous in *O. punctata* (listed as O.punctata1-6 for ease of representation) and #^4^ heterozygous in *O. sativa* (japonica as Osj1/Osj2), *O. sativa* (*indica* as Osi1/Osi2), *O. australiensis* (as O.australiensis1/ O.australiensis1) and *O. barthii* (as O.barthii1/O.barthii2). A single base pair insertion at the same position was observed in pre-*miR396c* sequences of both *O. punctata* and *O. officinalis* (highlighted in blue). (**B**)**:** Optimal Neighbour-joining tree generated using MSA data (MEGA11). Evolutionary distances were computed using the Maximum Composite Likelihood method, represented as the number of base substitutions per site. The genome type for each *Oryza* species is indicated within brackets.

*Mature miR396c sequence is conserved across most Oryza species* Pre-miRNA sequences from *Oryza* species for *miR396c* were retrieved from the NCBI database using BLAST. Since pre-miRNA sequences retrieved are reported overwhelmingly for diploid *Oryza* (AA) genomes^19^, we attempted to PCR amplify genomic stretches corresponding to the pre-miRNAs from both diploid (other than AA genomes) and tetraploid *Oryza* species belonging to other genotypes and also sought to validate data currently available for diploid AA *Oryza* genomes. The *Oryza* wild species in this study represents seven genome types (AA, BB, CC, EE, BBCC, CCDD and KKLL) of 10 representative genome types recognized by the OMAP resource^20^. The twelve *Oryza* genomes analysed in this study are listed in Table 1. Alignment of these sequences suggests that there is no variation in the mature miRNA regions of *miR396c* in the analysed *Oryza* species (with the exception of *O. coarctata*; Supplementary Fig. 1), though SNPs are observed in the pre-miRNA sequences (Fig. 1A & B).

**Table 1:** List of *Oryza* species used for the current study and accession numbers of precursor *miR396c* sequences.

Species (*Genome type*)	Source	Plant accession	Pre-*miR396c* Genbank accession no
*O. nivara (AA)*	CRRI, India	AC100010	ON622709
*O. glaberrima (AA)*	CRRI, India	AC37574	ON622704
*O. rufipogon (AA)*	CRRI, India	AC100028	ON622710
*O. sativa (japonica) (AA)*	CRRI, India	Dinorado/AC41038	ON622708
*O. sativa (indica) (AA)*	CRRI, India	IR20/AC41066	ON622705
*O. barthii (AA)*	CRRI, India	AC100498	ON622706
*O. punctata (BB)*	IRRI, Philippines	IRGC105690	ON596986
*O. alta (CCDD)*	IRRI, Philippines	IRGC105143	ON622703
*O. australiensis (EE)*	IRRI, Philippines	IRGC100882	ON622707
*O. minuta (BBCC)*	IRRI, Philippines	IRGC101141	ON622711
*O. officinalis (CC)*	IRRI, Philippines	IRGC100896	ON596987
*O. coarctata (KKLL)*	Pichavaram, India	MSSRF-1	ON596985

Underlined text provides a clickable link to the corresponding DNA sequence at the NCBI database https://www.ncbi.nlm.nih.gov/.

Seven SNPs and one indel are present in the stretch between the miRNA-miRNA* regions in the eleven pre-miR*396c Oryza* sequences examined. Among 7 SNPs detected, four SNPs were due to heterozygosity in selected *Oryza* species (Fig. 1A). A single base pair insertion at the same position was observed in pre-*miR396c* sequences of both *O. punctata* and *O. officinalis*. Halophytic wild rice *O. coarctata* pre-*miR396c* sequence is significantly different from *O. sativa* pre-*miR396c* and shows six base changes in the miRNA-miRNA* region (three each in miRNA and miRNA* regions; Supplementary Fig. 2). The pre-*miR396c* sequences from eleven *Oryza* species are 141–142 bp in length while for *O.* *coarctata* the pre-*miR396c* sequence is substantially reduced (133 bp). This 9 bp deletion falls within the intervening miRNA-miRNA* region. The *O. coarctata* pre-*miR396c* sequence reported here is identical to that present in the *O. coarctata* genome^21^ (Supplementary Fig. 2), suggesting *miR396c* function in halophytic wild rice may be significantly different.

### Pre-*miR396c *secondary structures from *Oryza* species can be divided into three groups

**Figure 2:**
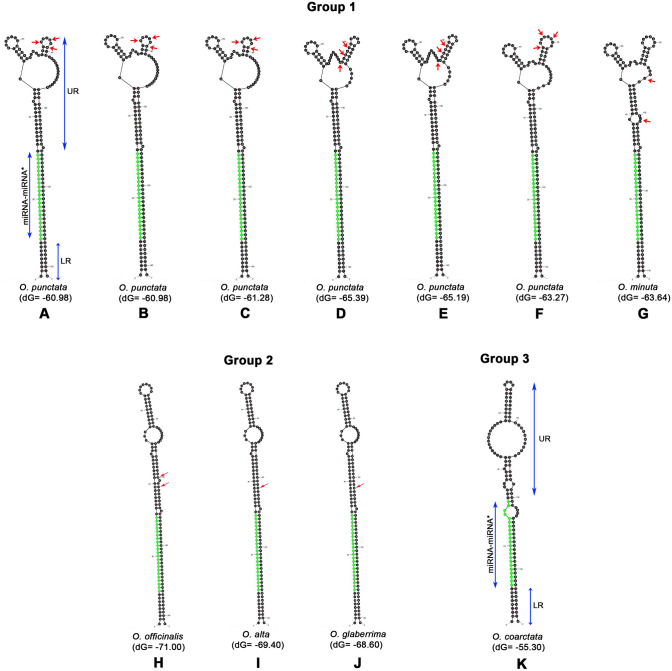
Predicted secondary structures for pre-*miR396c* using Mfold. Pre-*miR396c* structures from twelve *Oryza* species can be divided into three groups. Three elements are absolutely conserved across eleven *Oryza* spp*.* Pre-*miR396c* structures: (i) miRNA-miRNA* duplex (miRNA in green)(ii) a five bp bulge above the miRNA-miRNA* region (indicated by a grey box) (iii) 9 bp lower conserved region (LR) below the miRNA-miRNA* duplex (of which 6 bases show pairing). (**A**–**G**)**:** Group I contains pre-*miR396c* structures from *O. punctata* (142 bases) and *O. minuta* (141 bases). *O. punctata* pre-miRNA *396c* sequence shows heterozygosity at positions 74 and 79 (M = A or C) in addition to insertion of a single base at position 102 (indicated by red arrows; Fig. 2-1). (**H**–**J**)**:** Group II contains*. officinalis*, *O. alta* and O*. glaberrima* pre-*miR396c* structures differing only in an ‘A’ or ‘G’ at the 103 position of the pre-*miR396c* sequence (Fig. 2-2). (**K**)**:** Group 3 contains only *O*. *coarctata* pre-*miR396c*. The miRNA-miRNA* duplex region is indicated in green and perfect pairing is disrupted due to 6 SNPs.

The predicted secondary structure of pre-*miR396c* sequences from twelve *Oryza* species were examined vis-à-vis their sequence using Mfold^22^ (Fig. 2-1 and 2-2). Based on the secondary structures, the twelve pre-miRNA sequences were divided into three groups. Within group 1 and 2, certain pre-miR*396c* structural features are absolutely conserved. These include the miRNA-miRNA* regions (conserved across eleven *Oryza* species). In addition, a five bp bulge 3′ relative to the miRNA-miRNA* region and a 9 bp lower conserved region (LR) 5′ relative to the miRNA-miRNA* duplex are also found to be absolutely conserved in the eleven *Oryza* species. Group I contains pre-miRNA secondary structures from *O. punctata* and *O. minuta* that differ in length by one base (*O. punctata;* 142 bases; Fig. 2-1A–F). The *O. punctata* pre-miR*396c* sequence shows heterozygosity at positions 74 and 79 (M = A or C), in addition to insertion of a single base at position 102. As a result of heterozygosity at positions 74 and 79, variations in *O. punctata* pre-miR*396c* structures are observed that, in turn, result in variation of ΔG_folding_ from − 60.98 to − 65.39 kcals/mol (Fig. 2-1). These structures all show two conserved bulges in the Upper region (UR), including a Branched Terminal Loop (BTL) that differ slightly in their structure, contributing to variation in ΔG_folding_. The *O. minuta* pre-miR*396c* structure also shows a BTL in the UR (Fig. 2-1G). Further, the second bulge in the base paired region below the BTL in *O. minuta* has 8 bases and is shifted in its position relative to that in *O. punctata*.

Among the wild and cultivated *Oryza* species examined, six *Oryza* species showed identical sequences within Group 2 and only variation at position 103 (G or A); only G: *O. glaberrima*, only A (*O. officinalis, O. alta, O. nivara, O. rufipogon*); heterozygosity (G/A; *O. barthii, O. australiensis*) in the specific accessions examined. In the two cultivated Asian rice accessions examined, (*O. sativa* sub. *indica*; *O. sativa* sub. *japonica*), also falling within Group 2, position 103 showed heterozygosity (G/A) in the pre-miR*396c* sequences. In Group 2, the *O. officinalis* pre-*miR 396c* secondary structure has one additional bulge in the UR*; O. alta* and O*. glaberrima*, pre-*miR396c* structures show two bulges, two loops and three paired stems in the UR; (Fig. 2-2H-J). *O. alta* and *O. glaberrima* structures differ at position 103 (R = G or A) in the UR of the pre-*miR396c* sequence. The presence of an “A” at the 103rd position results in a perfect U_38_-A_103_ base pair while the presence of a ‘G’ at the 103rd position disrupts this base pairing, resulting in a minor increase in the ΔG_folding_ of the pre-*miR396c* by -0.8kcals/mol.

The *O. coarctata* pre-miR*396c* structure is unique and occurs in group 3 (Fig. 2-2K). It shows one bulge, three loops and three stems in the UR region. The *O. coarctata* pre-*miR396c* secondary structure in addition has other unique features: (i) the 6 base variation in the miRNA-miRNA* region results in a loop at the 3′ end of the mature miRNA sequence, (ii) near perfectly base paired LR (eight of nine bases paired), (iii) a G-U wobble base pair in the LR (iv) a 7 bp terminal loop and (v) a low folding energy (-55.3 kcals/mol) compared to other *Oryza* species.

**Figure 3:**
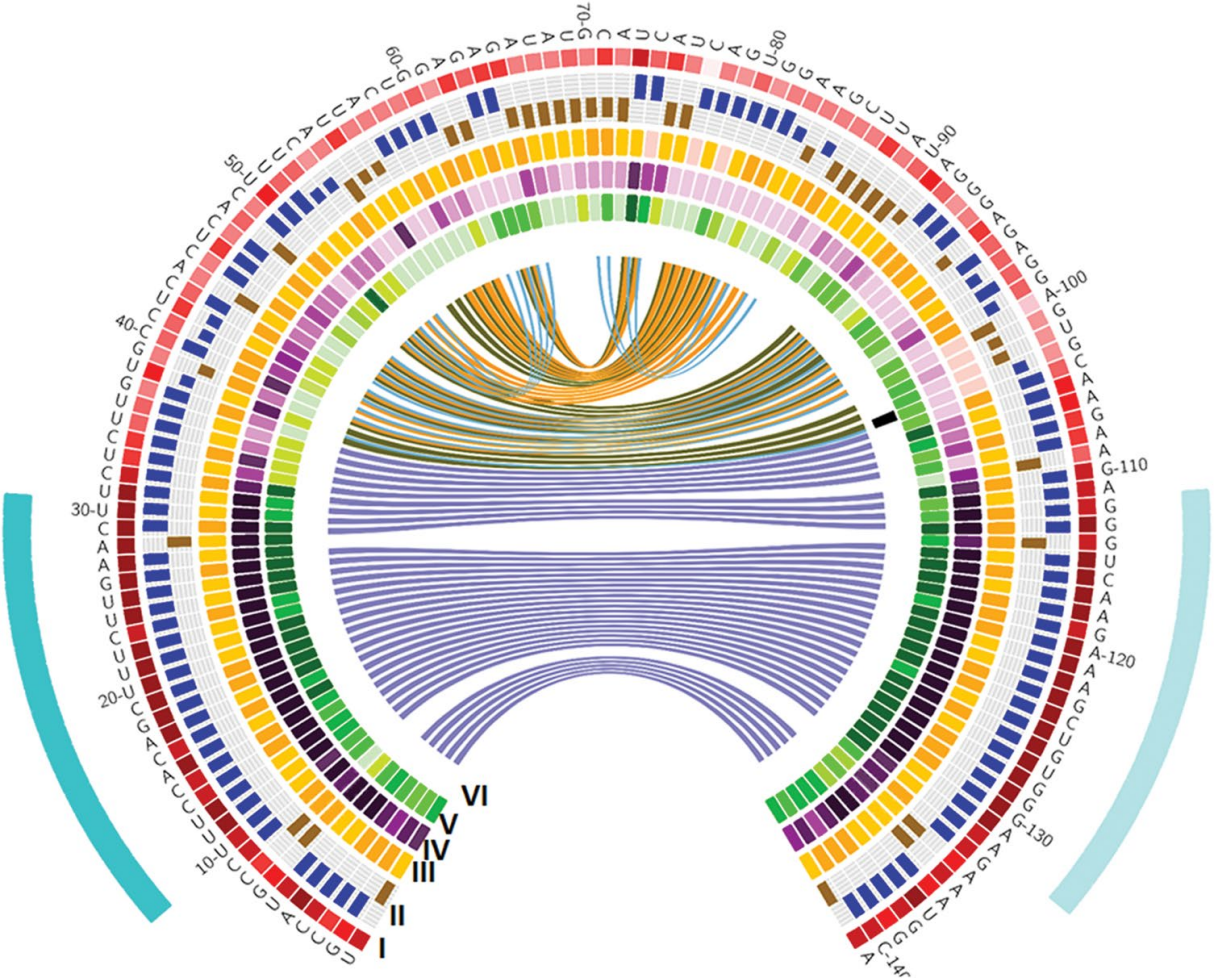
Circos analysis of sequence and structural conservation of pre-*miR396c Oryza*, dicot and monocot species. Ring I represents overall conservation of bases; ring II represents a histogram depicting frequency of base pairing (blue bars) and unpaired bases (brown bars) in *Oryza* species. Ring III represents conservation score for each base among O*ryza* species; darker colour indicates a higher conservation score (9) while a lighter colour indicates lower conservation score (1). Ring IV and Ring V represents the conservation scores observed for dicot and monocot species respectively. Ring VI represents the SNP observed in 3024*O. sativa* genomes.

A conserved SNP variation (A/G; rs10234287911) is present in pre-*miR396c* sequences within the genus *Oryza.* Pre-*miR396c* diversity across dicots, monocots and 3024 cultivated rice genomes was examined and is represented using a Circos plot (Fig. 3)^23^. The outermost ring is indicative of overall nucleotide sequence conservation across dicotyledonous and monocotyledonous species, with a darker shade indicating a higher conservation score. In case of pre-*miR396c* we have presented data for 74 precursors from 34 dicot species and 17 precursors from 7 monocot species. Ring II indicates the frequency and distribution of paired (blue bars) and unpaired bases (brown bars) for every position in the pre-miRNA precursor, with inner darker blue connecting ribbons representative of base pairing across *Oryza* species. The *O. coarctata* pre-*miR396c* sequence was not included in the Circos analysis because of a substantial variation in length vis-à-vis other *Oryza* species (133 bases vs. 141 bases). The division of the pre-*miR396c* sequences from *Oryza* species into three groups, based on secondary structure, is represented by orange (Group 1), dark green (Group 2) and light blue (Group 3) ribbons respectively. Rings III, IV and V represent pre-*miR396c* sequence conservation across *Oryza* species, dicotyledonous and monocotyledonous species respectively. Ring VI indicates nucleotide conservation of pre-*miR396c* sequences across 3024 cultivated rice *O. sativa* genomes. High conservation of the miRNA-miRNA* regions was observed in both dicots and monocots; further, high conservation was also observed in the lower stem region, preceding the miRNA-miRNA* duplex in dicots as well as monocots.

**Figure 4:**
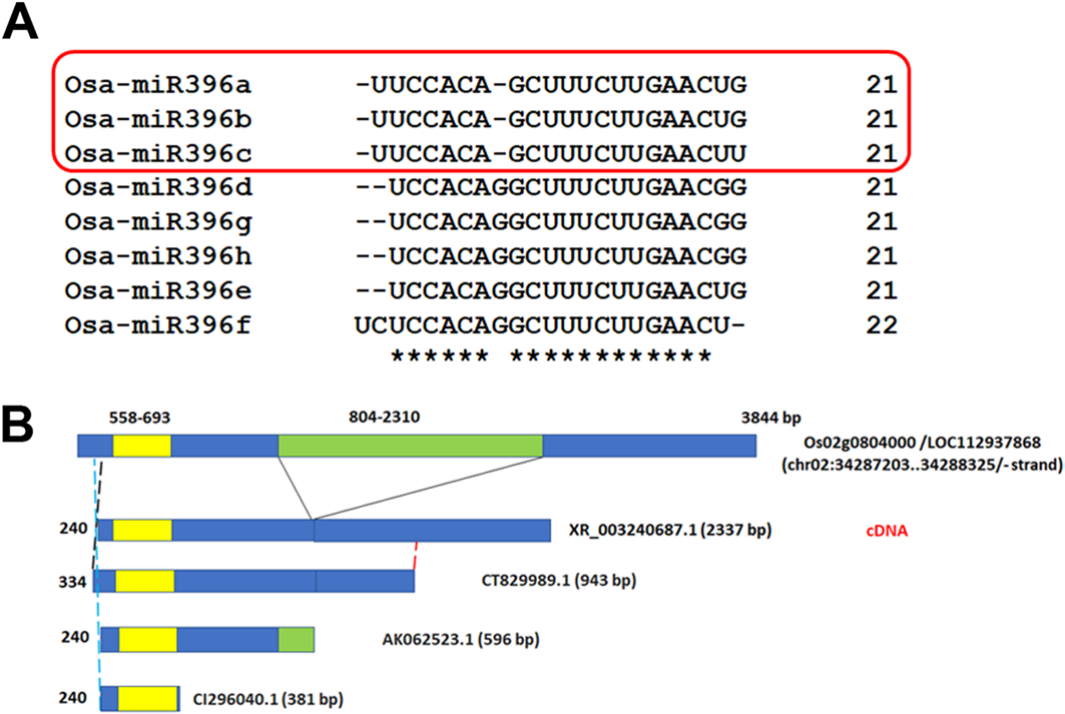
(**A**) Alignment of *miR396* mature sequences from *O. sativa*. Mature *miR396a-c* sequences group more closely (boxed in red) relative to miR396d-h. (**B**): Pictorial representation of the *miR396c* locus in *O. sativa* (*Os02g0804000*; negative stand; chromosomal coordinates indicated) with corresponding non-coding RNA (ncRNA; XR_003240687.1) and cDNA/EST sequences reported in the NCBI database. Exonic segments are indicated in blue, and the intronic region in green. The region marked in yellow in exon 1 corresponds to the pre-*miR396c* region. Numbers adjacent to dotted green, blue lines (5′ end) and red lines (5′ end) next to cDNA/EST sequences indicates corresponding base in the *miR396c* genomic sequence (*Os02g0804000*). Of these, cDNA (AK062523.1) shows partial intron retention.

As observed above for the wild and cultivated *Oryza* species in group 2, a single SNP variation (A/G; rs10234287911) corresponding to the same 103rd position in the pre-*miR396c* sequences was also observed in the examined 3024 *O. sativa* sequences (3K *O. sativa* rice genomes). This SNP variation occurs in the stem structure of the pre-*miRNA396c*, in the UR just above the miRNA-miRNA* paired region (Supplementary Fig. 3). As already mentioned, an ‘A’ at the 103rd position in the *O. sativa* pre-miRNA sequence results in a perfect U_38_-A_103_ base pair while the presence of a ‘G’ at the 103rd position disrupts this base pairing, resulting in a minor increase in the ΔG_folding_ of the pre-*miR396c* by  − 0.8 kcals/mol. In addition, the presence of A_103_ results in a contiguous stretch of seven base paired residues just above a five bp bulge in the upper region in contrast to the presence of G_103_, resulting in 5 base paired residues flanking U_38_-G_103_ and G_39_-U_102._ Of the 3024 *O. sativa* genomes examined, 2184 genomes had ‘G’ at the 103rd position while 740 *O. sativa* genomes had ‘A’; in addition, 83 *O. sativa* sequences were heterozygous for this allele in pre-*miR396c* (Supplementary Table 1), similar to the data obtained for heterozygosity in *O. sativa* (*indica* and *japonica*; R = A or G) as well as *O. barthii* and *O. australiensis* from our sequencing study. In addition, only a ‘G’ or ‘A’ was found at position 103 in all *Oryza* species except *O. coarctata*, and is suggestive of evolutionary selection for either an ‘A’ or a ‘G’ base at the 103rd position.

### Pri-*miR396c* in *O. sativa* shows complex post-transcriptional processing

Since *MIR396* is a multigene family in *O. sativa,* mature *miR396a-h* sequences were compared. Of these, *miR396a*-c showed near sequence identity, with mature *miR396c* differing from *miR396a/b* by one base (3' terminal U; Fig. 4A). This suggests that *miR396c* can be distinguished from *miR396a*/b using specific probes or primers. A noncoding RNA (XR_003240687.1) corresponding to *MIR396c* (*Os02g0804000*) was retrieved. In addition, cDNA/ESTs corresponding to the *O. sativa* pre-*miR396C* cDNA: AK062523.1; ESTs: CT829989.1, CI029080.1, CK053360.1, CK045291.1, CT856578.1, CT850193.1 and CI296040.1) were also retrieved. The genomic sequence of *Osa-MIR396c* (*Os02g0804000*) was aligned with the non-coding RNA (XR_003240687.1), showing the presence of two exons and an intervening intron (Fig. 4B). Of the cDNA/ESTs retrieved, only CT829989.1 showed correct splicing but retained only 132 bp as part of the second exon while other cDNAs/ESTs showed partial intron retention (Supplementary Fig. 4). The presence of intron retaining pri-*miR396c* isoforms in cultivated rice, *O. sativa*, suggests that *MIR396c* regulation and *osa-miR396c* biogenesis might be complex. Extending analysis to the entire *Os02g0804000* locus (inclusive of the intronic segment) led to the identification of 27 SNPs and 7 indels among 3024 rice accessions (pri-*miR396c* region, including intronic segment). The indels include a GA repeat region in the first exon (following the pre-*miR396c*) and a TA repeat region in the intron.

### Expression of *miR396c*, pre-*miR396c* and pri-*miR396c* in rice landraces under salinity and heat stresses

**Figure 5:**
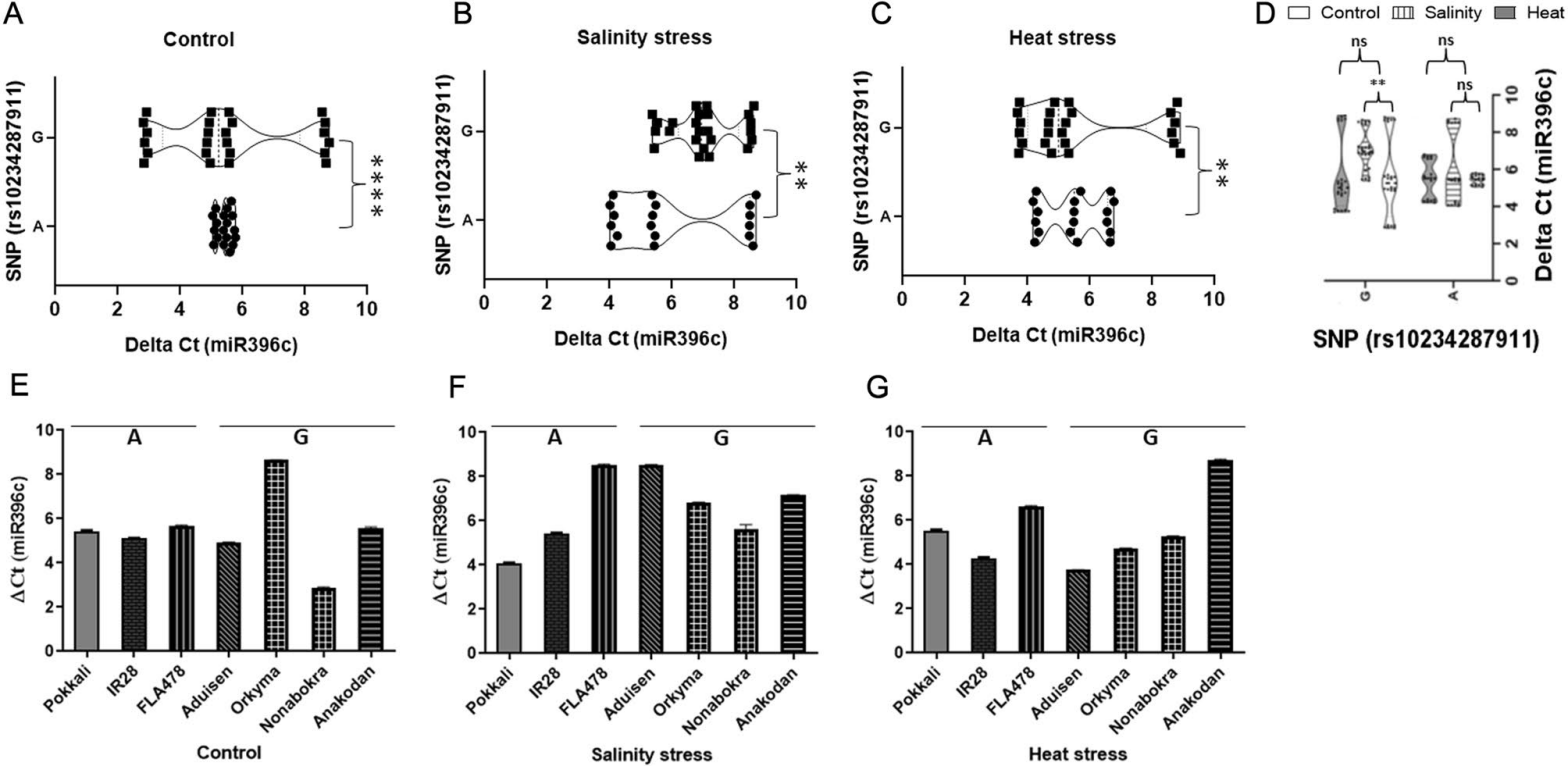
qRT-PCR analysis of mature *miR396c* expression under control (untreated), salinity and heat treatments in rice landraces/varieties grouped either ‘A’ or ‘G’. Violin plots representing ΔC_T_ values for *miR396c* expression under control conditions in (**A**), salinity in (**B**) or heat stress in (**C**) for landraces/varieties grouped either as ‘A’ or ‘G. In (**E**–**G**), ΔC_T_ values for *miR396c* expression for individual landraces under the same conditions is shown. Pairwise statistical analysis of *miR396c* expression in landraces/varieties grouped either ‘A’ or ‘G’ (**A**–**C**) or within landraces grouped ‘A’ or ‘G’ (control vs salinity or heat) using pairwise Student’s t-test (GraphPad V 6.0). Data is for three biological replicates per landrace/varieties per treatment (n = 3) with two/three technical internal replicates per treatment. ****P < 0.0001, **P < 0.01; ns: non significant.

**Figure 6:**
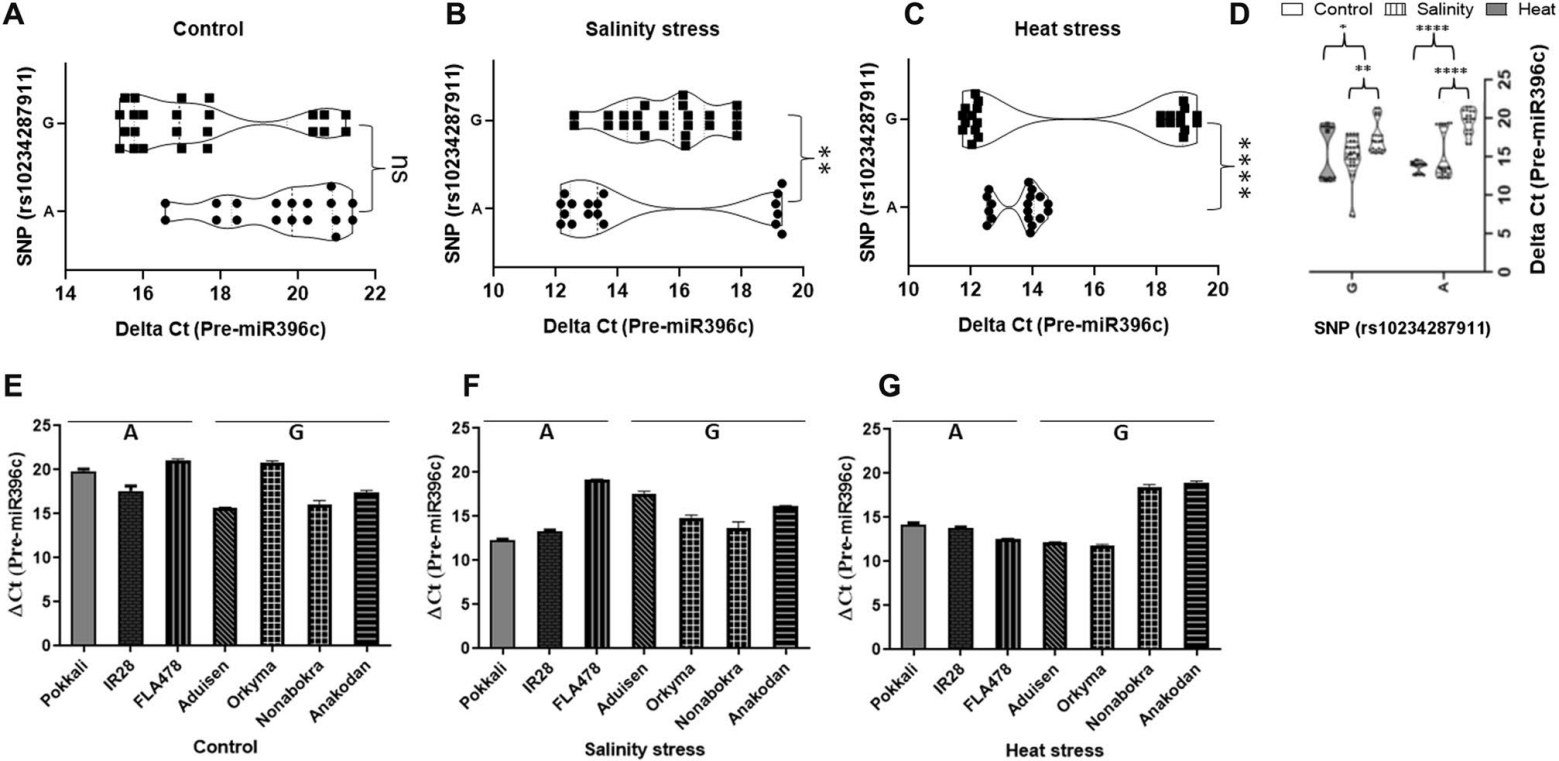
qRT-PCR analysis of pre-*miR396c* expression under control (untreated), salinity and heat treatments in rice landraces/varieties grouped either ‘A’ or ‘G’. Violin plots representing ΔC_T_ values for pre-*miR396c* expression under control conditions in (**A**), salinity in (**B**) or heat stress in (**C**) for landraces/varieties grouped either as ‘A’ or ‘G. In E–G, ΔC_T_ values for pre-*miR396c* expression for individual landraces under the same conditions is shown. Pairwise statistical analysis of pre-*miR396c* expression in landraces/varieties grouped either ‘A’ or ‘G’ (**A**–**C**) or within landraces/varieties grouped ‘A’ or ‘G’ (control vs salinity or heat) using pairwise Student’s t-test (GraphPad V 6.0). Data is for three biological replicates per landrace/varieties per treatment (n = 3) with two/three technical internal replicates per treatment. ****: *P* < 0.0001, **P < 0.01; *P* < 0.05; ns: non significant.

**Figure 7:**
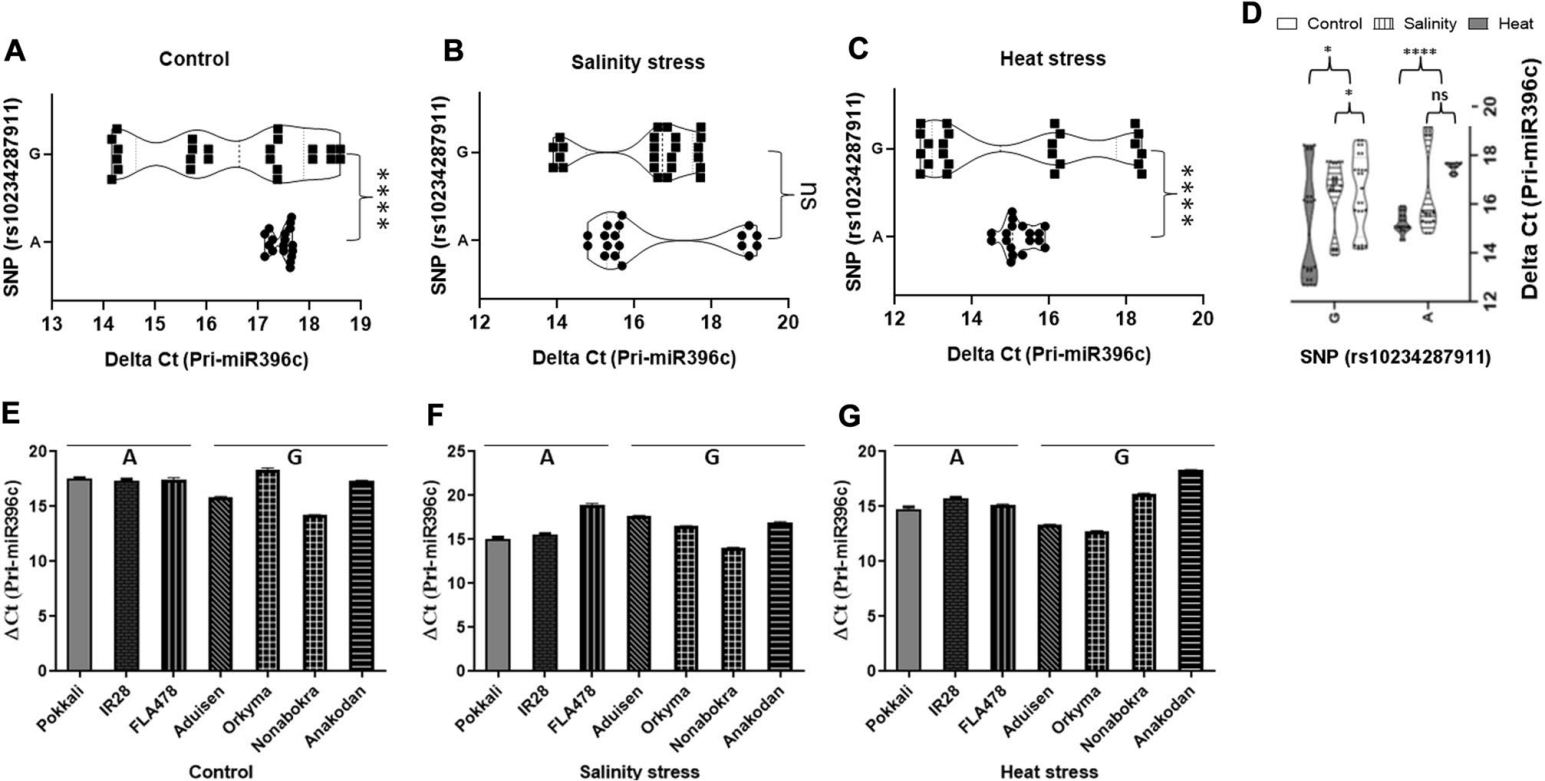
qRT-PCR analysis of pri-*miR396c* expression under control (untreated), salinity and heat treatments in rice landraces/varietiesgrouped either ‘A’ or ‘G’. Violin plots representing ΔC_T_ values for pri-*miR396c* expression under control conditions in (**A**), salinity in (**B**) or heat stress in (**C**) for landraces/varieties grouped either as ‘A’ or ‘G. In (**E**–**G**), ΔC_T_ values for pri-*miR396c* expression for individual landraces/varieties under the same conditions is shown. Pairwise statistical analysis of pri-*miR396c* expression in landraces grouped either ‘A’ or ‘G’ (**A**–**C**) or within landraces/varieties grouped ‘A’ or ‘G’ (control vs salinity or heat) using pairwise Student’s t-test (GraphPad V 6.0). Data is for three biological replicates per landrace/varieties per treatment (n = 3) with two/three technical internal replicates per treatment. ****: *P* < 0.0001, **; *P* < 0.05; ns: non significant.

**Figure 8:**
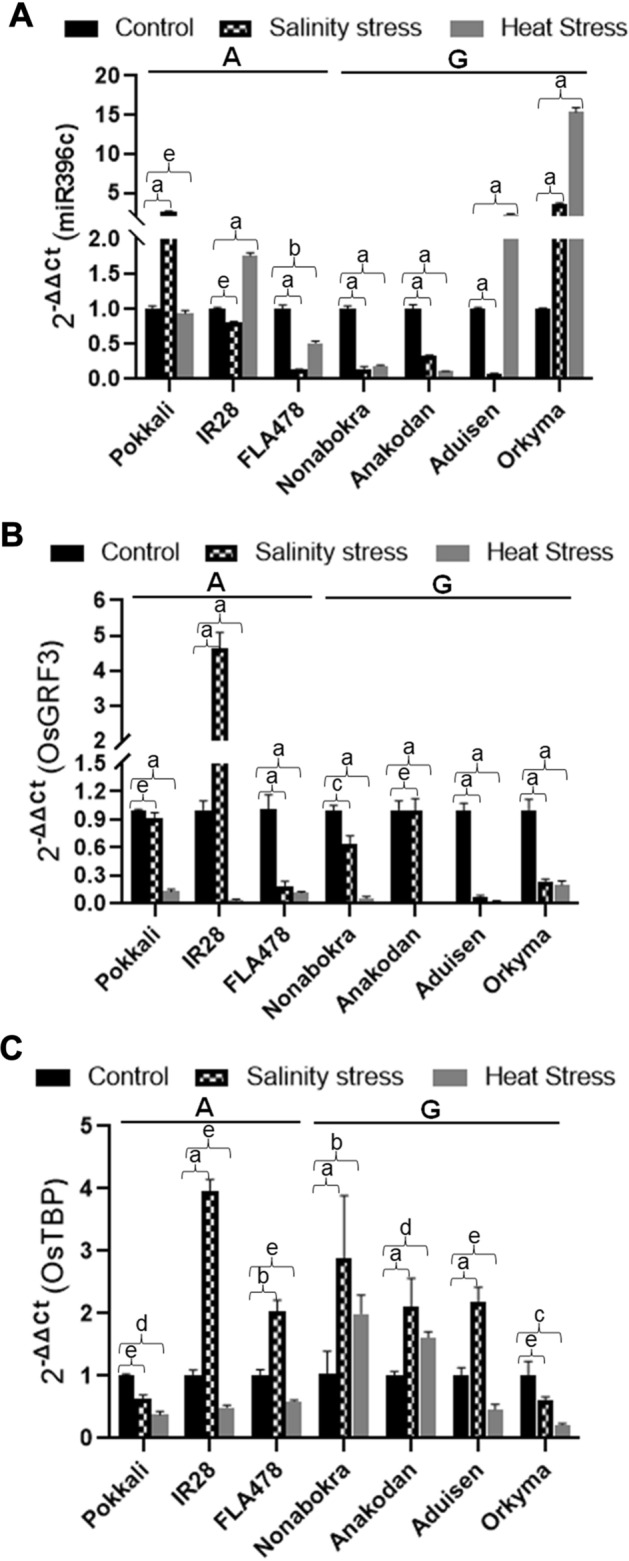
Expression analysis of (**A**) *miR396c* and target genes (**B**) OsGRF3 and (**C**) OsTBP under control, salinity and heat stress treatments in selected landraces/varietiesgrouped either ‘A’ or ‘G’. Pairwise statistical analysis of *miR396c or OsGRF3 or OsTBP* expression in landraces grouped either ‘A’ or ‘G’ (**A**–**C**) for each landrace/varieties (control vs. salinity or heat) using pairwise Student’s t-test (GraphPad V 6.0). Data is for three biological replicates per landrace/varieties per treatment (n = 3) with two/three technical internal replicates per treatment. a: *P* < 0.0001; b: *P* < 0.001; c: *P* < 0.01, d: *P* < 0.05; e: non significant.

The SNP variation (A/G; rs10234287911) identified in the *Oryza* spp. above was validated in additional 43 landraces from coastal regions of India^24^. PCR primers were designed to amplify a 141 bp fragment of pre-*miR396c* (Supplementary Fig. 5). Sequence analysis revealed that among 43 landraces, 22 showed the presence of an ‘A’ base at the 103rd position whereas 21 landraces had a ‘G’ at the same position. Thus, both bases appeared to be present at almost equal frequency in the examined landraces. Since this SNP variation occurred outside of the miRNA-miRNA* region, we examined if the variation may have a role in miR*396c* biogenesis from pre-miRNA. Over-expression of *miR396c* is associated with reduced salinity tolerance in rice^13^. Further, higher temperatures may alter pre-miRNA structure stability and hence *miR396c* abundance under heat stress (measured as *miR396c* expression) was examined. Therefore, we randomly selected seven landraces, three having either an ‘A’ (Pokkali, IR28 and FL478) and four with a ‘G’ (Nona Bokra, Anakodan, Aduisen and Orkyma) at the 103rd position in the pre-*miR396c* sequence and we examined *miR396c* expression in the landraces/varieties under salinity and heat stress conditions with appropriate untreated controls. Under control, salinity and heat treatments, *miR396c* expression in landraces/varieties grouped either ‘A’ or ‘G’ differed significantly as measured by statistical pair wise (Students t-test) analysis (Fig. 5A-C). *MiR396c* expression under salinity was increased significantly in landraces/varieties grouped ‘G’ while there was no significant difference in landraces/varieties grouped ‘A’ (Fig. 5D). Heat stress did not bring about a significant change in *miR396c* expression in landraces grouped either ‘A’ or ‘G’ (Fig. 5D). Further, landraces/varieties with an ‘A’ at the 103^rd^ position in the pre-*miR396c* sequences showed more tightly clustered expression under control conditions, while landraces/varieties with a ‘G’ at the 103rd position in the pre-*miR396c* sequences showed more distributed expression (Fig. 5A–C and E–G). Pre-*miR396c* expression under control (untreated), salinity and heat were also examined (Fig. 6A–G). Again, under salinity and heat treatments, pre-*miR396c* expression in landraces/varieties grouped either ‘A’ or ‘G’ differed significantly (highly significant under heat stress; Fig. 6A–C and E–G). Pre-*miR396c* expression was increased highly significantly under both salinity and heat stress in landraces/varieties grouped ‘A’ and to a lesser extent in landraces grouped ‘G’ (Fig. 6D). Significant positive correlation was seen between pre-*miR396c* and *miR396c* expression under control conditions in landraces/varieties grouped ‘G’ and under salinity treatment for landraces grouped ‘A’ (Supplementary Fig. 6A and B respectively). Finally, pri-*miR396c* expression under control (untreated), salinity and heat was also examined in the same set of samples (Fig. 7A–G). Pri-*miR396c* expression under control and heat treatments, differed significantly in landraces/varieties grouped either ‘A’ or ‘G’ but not under salinity (Fig. 7A–C and E–G). Further, pri-*miR396c* expression was significantly upregulated under heat stress in landraces/varieties grouped ‘A’ and to a lesser extent under both salinity and heat in landraces grouped ‘G’ (Fig. 7D). A significant correlation was found between pri-*miR396c* and pre-*miR396c* accumulation under control and heat stress conditions in landraces/varieties grouped ‘G’ and heat stress and salinity in landraces/varieties grouped ‘A’ Supplementary Fig. 6C–F). Expression of *miR396c* target genes (*OsTBP* and *OsGRF3*) in the same biological samples was examined under control, salinity and heat treatments. Overall, qRT-PCR data for the samples shows that the expression level of target genes was inversely correlated with *miR396c* expression and in most cases, statistically significant (Fig. 8A–C).

Small RNA northern blot analysis from landraces grouped ‘A’ or ‘G’, under control (Supplementary Fig. 7A), salinity and heat treatments (Supplementary Fig. 7B, C; Supplementary Fig. 12) did not show any differences in *miR396c* expression levels. Mature *miR396a* differs from *miR396c* at the terminal 3' base. However,* miR396a* expression levels are considerably lower as compared to *miR396c*, as longer autoradiographic exposure was required (5 days) to obtain a signal compared to *miR396c* (3 days; Supplementary Fig. 7A). Given that probes can occasionally cross-hybridize, RNA-seq data from^25^ was used to confirm expression level differences between *miR396a* and *miR396c* (Supplementary Fig. 8).

## Discussion

Plant miRNA precursors show high structural diversity and are processed by different modes to produce mature miRNAs ^3,4,20^. *Oryza* species span ~ 15 million years of evolution and the absolute conservation of mature miRNA sequences of *miR396c* (*O. coarctata* is an exception) in eleven *Oryza* genome types is suggestive of a strong evolutionary constraint to maintain its interaction with target genes in the genus *Oryza*.

Pre-*miR396c* sequences show absolute conservation of miRNA and miRNA* regions in most *Oryza* species examined. Based on the pre-*miR396c* structures, three distinct structural groups could be recognized, with *O. coarctata* pre-*miR396c* forming a separate group. As mentioned previously, three elements were absolutely conserved across the eleven *Oryza* pre-*miR396c* structures (miRNA/miRNA* regions, 5 base bulge 3′ to miRNA/miRNA* region and 9 bp stem in LR) and may be indicative of base to loop pre-miRNA processing, similar to *miR172a*^4^. Group 1 members show the presence of a branched terminal loop (BTL). BTLs seen in *Arabidopsis* pri-*miR166* alter processing direction from a base-to-loop mode to a bidirectional mode that has implications on miRNA abundance^26^. BTLs reduce the efficiency of *Arabidopsis miR157c* processing and replacement by a 4 nucleotides loop increases processing efficiency^27^. Group 2 members show a common conserved 10 bp terminal loop (TL) in the UR. In animal systems, the primary miRNA transcript is processed to pre-miRNA by Drosha which is then processed by Dicer to generate the mature miRNA-miRNA* duplex. Around 14% of all human pri-miRNAs have conserved terminal loops that are indicative of a requirement to provide a docking site for auxiliary factors that govern pri-miRNA processing^28^. Lin28 (abnormal cell lineage factor 28) interacts with the miRNA precursors of the *let-7* family, by recognizing different elements of the TLs of miRNA precursors while multifunctional KSRP [KH (K-homology) splicing regulator protein] interacts with pre-*let-7a*, recognizing a G-rich site in the TL^29^. The presence of the conserved 10 bp loop sequence in eight *Oryza* species is strongly suggestive of its recognition as an important element in the pre-*miR396c* processing machinery. In *O. coarctata* this 10 bp stretch is absent and may have different miRNA processing from other *Oryza* species.

The comparatively higher ΔG_folding_ of *O. coarctata* pre-*miR396c vis-à-vis* other *Oryza* species may have implications for processing. In *Arabidopsis*, allelic variation in the *miR824*-encoding locus favours a thermal resistant substructure in the precursor that impacts processing of mature *miR824*^30^. In other O*ryza* species examined, the 9 bp LR shows 6 paired bases, while in *O. coarctata* 8 of 9 bases shows pairing that also includes a unique U-G wobble. This U-G wobble arises due to an SNP in the LR just below the miRNA* region. Directed substitutions and variation analysis of *Arabidopsis MIR390a* and *MIR390b* loci shows that base pair properties and nucleotide identity 4–6 bases below the *miR390*/miR390* duplex region contributes to the efficiency and accuracy of precursor processing^31^. *O. coarctata* pre-*miR396c* shows unique ten bp loop within the miRNA-miRNA* region (towards the 3′ end of the mature miRNA sequence). This loop arises due to two SNPs in the mature miRNA and 2 SNPs in the miRNA* 396c sequence at 3′ end. *A. thaliana* miRNA:miRNA* duplexes feature a high number of natural polymorphisms that can affect base pairing and thus reduce accumulation of mature miRNAs^32^. The 3 SNPs in the putative miRNA region of *O. coarctata miR396c* are also likely to alter target specificity.

*miR396c* in *O. sativa* can be distinguished from other family members due to sequence specific differences or expression level variation. This has been observed in this study as well as others^33,34^. SNPs in pri-miRNA, pre-miRNA and mature miRNA sequences can potentially affect the maturation of miRNAs, their expression level and base pairing at the target site respectively, each mechanism in turn controlling/contributing to gene regulation. Predominantly, polymorphisms in pre-miRNA can influence miRNA maturation and thereby regulate miRNA expression^35^. We identified a G/A SNP variation (rs10234287911) at the 103rd position of *O. sativa* pre-*miR396c* examined sequences that potentially disturbs an A-U base pair if G replaces the A, resulting in the introduction of a G-U wobble pair destabilizing a perfectly base paired stem region that follows the miRNA-miRNA* region and a minor decrease in the ΔG_folding_ of the pre-miRNA 396c by 0.8 kcals/mol (Supplementary Fig. 3). Mutations that destabilize the region below the terminal loop in *Arabidopsis* precursor *miR172a* resulting in an open structure abolishes *miR172* expression while mutations that stabilize the structure of the loop do not affect *miR172a* biogenesis^36^. An SNP in pre-*miR1666* decreases mature miRNA expression and is associated with chicken growth traits^37^. Structurally different alleles of *Arabidopsis* precursor *miR824* likely show differential processing of mature *miR824* in *A. thaliana*^30^. Interestingly, of the twelve sub-populations recognised in the sequenced rice genomes^18^, four clusters including East Asian temperate (GJ-tmp), Southeast Asian Subtropical (GJ-sbtrp), South East Asian tropical (GJ-trp) and admix (GJ-admix) categories showed a clear 100% presence of the major ‘G’ allele. The ‘G’ allele also predominates in six other recognized categories in the same data set. On the other hand, the minor allele ‘A’, predominates in two groups containing Aus, Boro and Rayada ecotypes from Bangladesh and India (circum Aus group-cA) and the Basmati and Sadri aromatic varieties (circum Basmati group-cB) (Supplementary Fig. 9; Supplementary Table 1). The minor allele ‘A’ occurs in samples taken from South/South East Asian countries (Supplementary Fig. 9; Supplementary Table 2) while the major allele appears to predominate in South-East Asian countries (samples from India, China occurs in equal numbers in both categories). More precise passport data for the samples might help to correlate more precisely the geographic coordinates with geographical distribution of the SNP.

The G/A SNP variation occur at almost equal frequencies in the 43 landraces/varieties examined. Further, a significant correlation and distinction appears to exist between the two groups (G/A) vis-à-vis expression levels of the mature *miR396c* in leaf tissues of *O. sativa* landraces/varieties under control salinity or heat stress. This, in turn, impacts expression of target genes *OsTBP* and *OsGRF3*. Further, *miR396c* expression under salinity is downregulated in most landraces/varieties and as reported previously by^13^. However, only under salinity there is a significant *miR396c* expression change in the landraces grouped G. Given the association of *miR396c* with salinity stress in rice, it may be that specific factors control *miR396c* levels by pre-*miR396c* processing under salinity but not heat stress. Many regulatory proteins influencing the folding, stability, and/or processing of pre-miRNAs have been identified and described^2,38^ suggesting that pre-miRNAs are modified, folded, and processed co-transcriptionally^39^. More recently, it has been suggested secondary structural features of precursor miRNAs may have a role in not only controlling precursor miRNA processing, but also RISC loading efficiency onto AGO1^40,41^. SNPs in *Arabidopsis* miRNAs (ecotypes) that are predicted to have subtle effects on pre-miRNA secondary structure mostly occur in the double-stranded stem regions^42^. Further, if the SNPs have structural effects, these were found to be subtle and did not affect general integrity of the stem-loop structure. This may be similar to the SNP identified in this study. However, Northern analysis of landraces/varieties grouped ‘A’ or ‘G’ under the same set of conditions did not reveal significant expression level differences. Overall, miRNA quantification methods differ in specificity and accuracy^43^.

Extending expression analysis to pre-*miR396c*, significant differences were observed between landraces/varieties grouped ‘A’ or ‘G’ under salinity and heat. For pri-*miR396c* significant differences were observed between landraces/varieties grouped ‘A’ or ‘G’under control and heat stress conditions. The promoter region of the *MIR396c* locus (*Os02g0804000*) is enriched in ABRE elements and has TC-rich regions^13^. Further both pri-*miR396c* and pre-*miR396c* expression are upregulated in most landraces/varieties examined under heat. Also, pre-*miR396c* expression is upregulated under salinity in most landraces but *miR396c* expression is downregulated in most landraces/varieties under salinity (Pokkali and Orkyma are exceptions). There may thus be mechanisms controlling *miR396c* biogenesis in rice under salinity in leaf tissues. A partial intron retaining *O. sativa miR396c* cDNA (pri-*miR396c*; AK062523.1; Fig. 4), when constitutively overexpressed in *Agrostris stolonfera* (creeping bentgrass), is processed correctly to give mature *miR396c* and is associated with stunted growth but increased salinity tolerance^44^. Further, transgenic bentgrass lines bypass a vernalization requirement for flowering^45^. Thus, intron retaining pri-*miR396c* transcripts appear to be functional. The above data suggests complex transcriptional and post-transcriptional (including intron retention) mechanisms of *miR396c* regulation in rice.

The conservation of SNP rs10234287911 in the pre-*miR396c* sequence in wild as well as cultivated *Oryza* species suggests a possible role in mature miRNA biogenesis. Replacing the G/A hypothetically with either C or T results in *miR396c* secondary structures with significantly increased free energy values and lowered stability (Supplementary Fig. 4). Further, analysis of the 3000 rice genome data shows (i) the predominance of one allele (G) in the analysed population, (ii) exclusive or near exclusive presence in certain subpopulations of the major allele (Supplementary Fig. 9) and the pre-dominance of the minor allele in Aus and aromatic sub-populations (Supplementary Fig. 10). The pre-miRNA structure with the major ‘G’ allele shows less structured base pairing in the region closer to the terminal loop while minor ‘A’ allele has a more stable structure. MiRNA maturation efficiency may be governed by dynamic structural transitional states of pri- and precursor miRNAs (including 5′ UG/GU 3′ wobble pairs), adding another possible layer in regulation of miRNA biogenesis in response to environmental or developmental cues^43,46^. We hypothesize that the more stable structure conferred by the ‘A’ allele may be processed more readily vis-à-vis the structure conferred by the ‘G’ allele (with 5′ UG/GU 3′ wobble pairs). This may be reflective of the distribution pattern of mature *miR396c* expression levels in the two allele types. Agroinfiltration of cloned of *O. sativa* pre-*miR396c* structures with G_103_ or A_103_ variation(s) would help in determining conclusively the role of this SNP, if any, in controlling mature *miR396c* abundance.

## Conclusion

We present for the first time, pre-miRNA sequence data and secondary structural characteristics for *miR396c* across *Oryza* species. Conserved structural determinants within precursor *miR396c* sequences suggest a conserved base-to-loop mode of miRNA biogenesis. Pre-*miR396c* from halophytic *O. coarctata* shows substantial variation in length, sequence of precursor as well as miRNA/miRNA* regions that can impact biogenesis and target specificity. Further, a SNP identified in the precursor region of *miR396c* sequences (3024 *O. sativa* genomes) may have implications for miRNA abundance and target gene regulation under salinity but not under heat stress.

## Materials and methods

All methods were performed in accordance with the relevant guidelines and regulations.

### Genomic DNA isolation and primer design

*O. sativa miR396c* pre-miRNA sequence (MI0001048) of was obtained from miRbase^47^ and used as query sequence to retrieve corresponding plant pre-miRNAs from *Oryza* species (non-redundant; whole genome shotgun; genome survey sequence) present in the NCBI database using BLAST. In order to isolate the corresponding pre-miRNA sequences from other *Oryza* species (not reported so far), primers were designed to amplify the corresponding pre-miRNA from genomic DNA samples. DNA was isolated using CTAB from young leaf tissues of twelve *Oryza* spp.^48^. *Oryza* species used for PCR amplification of pre-miRNA sequences are listed in Table 1 along with source data. All necessary permissions were taken to obtain the samples.

### PCR amplification and sequencing of pre-miRNAs from *Oryza* species

PCR amplification was carried out in a reaction volume of 10 µl containing 50 ng genomic DNA template, 0.5 μM forward and 0.5 μM reverse primers (Table 2), Ampliqon Taq DNA Polymerase Master Mix RED (2x). Reaction conditions were as follows: initial denaturation (94 °C; 5 min), 35 cycles of denaturation (94 °C; 30 s), annealing (53 °C; 30 s), extension (72 °C; 30 s), with a final extension step at 72 °C (7 min) in a GeneAmp PCR System 9700 Thermal cycler (Applied Biosystems, California, USA). The amplified products were separated by agarose gel electrophoresis (1.5%), PCR products eluted using a gel purification kit (Favor Gen) and sequenced from both ends (Eurofins, India). The chromatogram data (forward and reverse sequences) obtained was analysed for base calling accuracy and a consensus sequence was determined for each PCR product using DNA Baser V 4.36. The sequences were aligned using Clustal Omega and analysed for variations^49^.

**Table 2:** Free energy of folding (ΔG_folding_) for the secondary structure of pre-*miR396c.*

S. No	Group I	ΔG_folding_
1	*O. punctata*1 *(BB)*	− 60.98
2	*O. punctata*2 *(BB)*	− 60.98
3	*O. punctata*3 *(BB)*	− 61.28
4	*O. punctata*4 *(BB)*	− 65.39
5	*O. punctata*5 *(BB)*	− 65.19
6	*O. punctata*6 *(BB)*	− 63.27
7	*O.minuta (BBCC)*	− 63.64

	Group II	ΔG_folding_
8	*O. officinalis (CC)*	− 71
9	*O. alta (CCDD)*	− 69.4
10	*O. glaberrima (AA)*	− 68.6
11	*O.nivara (AA)*	− 69.4
12	*O. rufipogon (AA)*	− 69.4
13	*O. australiensis*1* (EE)*	− 69.4
14	*O. australiensis*2* (EE)*	− 68.6
15	*O. barthii* 1*(AA)*	− 69.4
16	*O. barthii* 2*(AA)*	− 68.6
17	*O. sativa (indica)* 1*(AA)*	− 69.4
18	*O. sativa (indica)* 2*(AA)*	− 68.6
19	*O. sativa (japonica)* 1*(AA)*	− 69.4
20	*O. sativa (japonica)* 2*(AA)*	− 68.6

	Group III	ΔG_folding_
21	*O. coarctata (KKLL)*	− 55.3

### Secondary structure prediction of pre-*mir396c* from *Oryza* species

The pre-miRNA sequences obtained through sequencing were analysed using Mfold^22^ using default parameters. The folding temperature was fixed at 37 °C. The predicted structures were analysed and grouped manually based on the number of bulges, stem and loops.

### Analysis of variation in pre-*miR396c* sequence in *Oryza* species, dicots and monocots

Pre-miRNA sequences corresponding to *miR396c* from monocotyledonous and dicotyledonous species were retrieved from miRbase. Pre-miRNA sequences corresponding to the 3000 rice genomes were also retrieved^50^, ^18^ (https://snp-seek.irri.org/). ConSurf V 3.0 was used for predicting nucleotide conservation scores^51^, with the number with the highest value representing the most conserved base. Conservation in pre-miRNA sequences is represented using Circos^23^. The SNP data was downloaded from Rice SNP-Seek Database (https://snp-seek.irri.org/) as an Excel file and sorted based on subpopulations and the SNP. This data was manually fed into the ArcGIS software to generate map based data. Since the pre-*miR396c* is located on the reverse strand on rice chromosome 2 (coordinates), the data obtained from Rice SNP-Seek Database (a single SNP C/T at position 10,234,287,911) refers to the ‘non-coding’ strand (reference genome information IRGSP v 2.0) with reference to the pre-*miR396c* sequence. The corresponding sequence in the ‘coding strand of pre-*miR396c* would be G/A and is referred to as such throughout the study (map data however corresponds to the reference strand IRGSP v 2.0).

### PCR amplification and sequencing of pre-*mi96c* from *Oryza* sativa landraces

Pre-*miR396c* sequences were PCR amplified from genomic DNA of *O. sativa* rice landraces (Table 3; includes source data) and sequenced from both ends (Eurofins, India). All necessary permissions were taken to obtain the samples. Chromatogram data (forward and reverse sequences) obtained was analysed for base calling accuracy and a consensus sequence was determined for each PCR product using DNA Baser V 4.36.

**Table 3:** List of rice landraces used for SNP validation and accession numbers of precursor *miR396c* sequences. Landraces used in this study are according to^25^.

Landrace	*Pre-miR396c* Accession no	Landrace	*Pre-miR396c* Accession no
1. Nona Bokra-1	ON596942	24. Chettivirippu	ON596965
2. Nona Bokra-2	ON596943	25. Pallipuram Pokkali	ON596966
3. Nona Bokra-3	ON596944	26. Kagga	ON596967
4. Altaluti	ON596945	27. Korgut	ON596968
5. Nona Bokra-3 (IRRI)	ON596946	28. Kalamocha	ON596969
6. Hoogla	ON596947	29. Matla-1	ON596970
7. Katrangi	ON596948	30. Matla-2	ON596971
8. Darsal	ON596949	31. Dudheswar	ON596972
9. Rupsal	ON596950	32. Aduisen-1	ON596973
10. Kaksal	ON596951	33. Aduisen-2	ON596974
11. Marisal	ON596952	34. Kaatuponni	ON596975
12. Gheus	ON596953	35. Talmugur-1	ON596976
13. Jingasal	ON596954	36.Talmugur-2	ON596977
14. Nona Soren	ON596955	37.Talmugur-3	ON596978
15. Kalonunia	ON596956	38. Pokkali-2	ON596979
16. Patnai-23	ON596957	39. Pokkali-3	ON596980
17. Mundon-1	ON596958	40. Pokkali-4	ON596981
18. Mundon-2	ON596959	41. FL478	ON596982
19. Kamini	ON596960	42.IR28	ON596983
20. Hamilton	ON596961	43.IR29	ON596984
21. Anakodan	ON596962		
22. Orumundakan-1	ON596963		
23. Orumundakan-2	ON596964		

Underlined text provides a clickable link to the corresponding DNA sequence at the NCBI database https://www.ncbi.nlm.nih.gov/.

### Mining pri-*miR396c* sequences from databases

*O. sativa miR396c* (*Osa-miR396c*) sequence was used to query the NCBI (nr and EST) databases using BLAST. Sequences were aligned using Clustal Omega and cDNA and genomic sequence exon–intron boundaries defined using Splign [https://www.ncbi.nlm.nih.gov/sutils/splign/splign.cgi]. Primers corresponding to *O. sativa* pri-*miR396c*, pre-*miR396c* (as defined by miRbase) were designed based on this sequence information and used for qRT-PCR.

### Salinity and heat stress treatments

Four landraces with ‘A’ at the 103rd position in the expected PCR fragment (Supplementary Fig. 3 for reference; *O. sativa* ssp. *indica* cv. Pokkali, IR28, FL478) and four other landraces with ‘G’ nucleotide at the 103rd position (*O. sativa* ssp. *indica* cv. Nona Bokra, Anakodan, Orkyma and Aduisen) were selected randomly from the sequenced landraces. The plants were grown in controlled conditions (25 ± 2 °C; 12/12-h light/dark photoperiod; and 60% moisture) and transferred to the temperature and humidity controlled green house after 20 days. Salinity stress was imposed in hydroponics as described in^52^. Salinity stress was imposed using 100 mM NaCl (2 days) Similarly, the selected landraces with ‘A’ or ‘G’ at the 103rd position, grown in controlled conditions as described previously were also subjected to heat stress, incubated at 42 °C for 2 days. Parallel control samples were maintained at 25 ± 2 °C. Leaf tissues were harvested and frozen in liquid nitrogen.

### Total RNA Isolation and RT-qPCR

MiRNA was isolated using ReliaPrep miRNA Cell and Tissue Miniprep Kit (Promega, India) using manufacturer’s instructions and quantified using Thermo Scientific, Multiskan GO v 3.2. First strand cDNA was synthesized from total miRNA (2.5 μg) using a cDNA synthesis kit (Superscript III; Invitrogen, USA) following manufacturer’s instructions and stem loop primers^53^. STEM-LOOP RT-qPCR was performed as described in^54^ and expression of Osa-*Pri-miR396c*, Osa-*Pre-miR396c* and mature Osa-*miR396c* were quantified in the same sample with specific primers (Table 4) as described in^54,55^ using SYBR green chemistry (Quant Studio 6 Flex, Applied Biosystems, USA) *O. sativa* U6 SnoRNA (*Osa-U6*) served as housekeeping control for *O. sativa* pri-*miR396c*, pre-*miR396c* and mature *miR396c*^56^. The relative expression ratio for pri, pre and mature miRNA396c was determined using the ∆CT and ∆∆CT methods for three biological replicates, each containing three seedlings (each replication was checked in triplicate) and significance estimated using Student’s t-test (GraphPad Prism 6). Candidate gene expression (*OsGRF3*, *OsTBP*) was tested for the same set of salinity stress samples for which STEM-LOOP RT-qPCR was carried out. *Oryza eukaryotic initiation factor 4-α* (*eIF4-α*) and *actin* (*ACT*) served as housekeeping controls^54^. cDNA was prepared using the cDNA synthesis kit (Superscript III; Invitrogen, USA) following manufacturer’s instructions and qRT-PCR carried out using SYBR green chemistry. Primers used for this study are listed in Table 4.

**Table 4:** Primers used for amplifying *miR396c* from *Oryza* species and qRT-PCR.

PCR primer	Forward primer (5′ to 3′)	Reverse primer (5′ to 3′)
*miR396c* (genomic DNA template)	GGGCACCAAATTAAGTAGA	GTTGCAATGTGCATTGGATG

qRT-PCR primers	Forward primer (5′ to 3′)	Reverse primer (5′ to 3′)
Osa-Pri-miR396c	AGCCTGCAGATCTCGATCGA	GCTTCCACTGATGATGCATATCTC
Osa-Pre-miR396c	CCATGCCTTTCCACAGCTTT	CTCTTCTTGCACTCCTCTCCCTAT
OsmiR396c	CGCGCTTCCACAGCTTTCTTG	CCAGTGCAGGGTCCGAGGTA
osa-U6	TACAGATAAGATTAGCATGGCCCC	GGACCATTTCTCGATTTGTACGTG
OsTBP	CACGGCCTCTTTTGAGGAGTA	CTGGCATGGGAGCTGAAGTC
OsGRF3	GAGGGAGCCATTGTCATTCTTC	AAGGTGGCAAGGCTGTTGTC
eIF4-α	TGCCCAGCAAATTGAAAAGG	TGCACGCCACTAGCAAGAAT
Actin	TGATTGCACCACCAGAAAGAAA	TGCCAGGACCAGATTCATCAT
**Stem loop RT primer (5′ to 3′)**	GTCGTATCCAGTGCAGGGTCCGAGGTATTCGCACTGGATACGACAAGTTC	

### Total RNA Isolation, small RNA Northern Hybridisation and Small RNA-seq data analysis

The leaf samples treated as mentioned above were ground in liquid nitrogen and total RNA was extracted using TRIzol (Invitrogen) as per the manufacturer’s instructions. RNA and quantified using RNA BR dye (Thermo Fisher Scientific) in a Qubit Fluorimeter. Northern hybridisation (small RNA) was performed according to^57,58^. Briefly, 10 µg of total RNA was electrophoresed in denaturing acrylamide gel [15% with 8 M Urea; 19:1 acrylamide: bis-acrylamide ratio; at 80 V (3 h)]. RNA was semi-dry blotted onto a Hybond N^+^ membrane (GE Healthcare) at 10 V overnight (4 °C) and crosslinked to the membrane using UV(UVP Crosslinker)]. Hybridisation in UltraHyb-Oligobuffer (Ambion) with ^32^P end-labelled oligo probes (*miR396a*: 5′ CAGTTCAAGAAAGCTGTGGAA 3′ or *miR396c*-5p: 5′ AAGTTCAAGAAAGCTGTGGAA 3′) was performed at 35 °C for 12 h. Probes were end labelled using T4 Polynucleotide Kinase (NEB) as per manufacturer’s instructions with labelled γ-^32^P-ATP (BARC,India) and purified using G-25 columns (Illustra Microspin; GE Healthcare) Following hybridization, the blot was washed twice with 2 × SSC, 0.5% SDS (30 min each at 35 °C).The blots were exposed to phosphor screen (GE Healthcare) and imaged using a Phosphorimager (Typhoon molecular imager; GE Healthcare). The blots were stripped at 80 °C and re-probed with end labelled *U6* probe (equimolar mix of 5′ GGCCATGCTAATCTTCTCTGTATCGTT 3′ and 5′ GGCCATGCTAATCTTCTCTGTATCGTT 3′). For estimation of *miR396a*, *miR396c* expression levels, Reads per Million (RPM) values (two replicates each; three tissues; three *O. sativa* accessions and two wild *Oryza species*) from^25^ were used.

## Supplementary Information

Supplementary Information 1.

Supplementary Information 2.

Supplementary Information 3.

## Data Availability

All sequence datasets generated and/or analysed during the current study are available at NCBI (*O. sativa* cultivated rice landraces pre-*miR396c* Accession numbers: ON596942-ON596984, Table 3; wild rice pre-*miR396c* Accession numbers: ON596985-ON596987; ON622703-ON622711; Table 1).

## Abbreviations

EC: Electrical conductivity
SNP: Single nucleotide polymorphism
