# Supplementary material for: A conserved SNP variation in the pre-miR396c flanking region in Oryza sativa indica landraces correlates with mature miRNA abundance
Source: Sci Rep. 2023 Feb 7;13:2195. doi: 10.1038/s41598-023-28836-1 (PMC9905475; doi:10.1038/s41598-023-28836-1)
Supplement: Supplementary file 1 — Supplementary Information 1. [file 41598_2023_28836_MOESM1_ESM.pdf]

Supplementary Figure 1: Pre-*miR396c* sequences from twelve *Oryza* species. Sequences with heterozygous positions for each species have been listed individually. Bases indicated in red show heterozygosity.

GROUP 1:
>O.punctata1_M74(A)_M77(A)_R79(A)
TGCCATGCCTTTCCACAGCTTTCTTGAACTTCTCTTGTGCCTCACTCACTTTCATTACT
GGAGAGATATGCAT**A**AT**A**A**A**TGGAAGCTTATAGGGAGAGGAGTAGCAAGAAGAGG
GTCAAGAAAGCTGTGGGAAGAAATGGCA
>O.punctata2_M74(A)_M77(C)_R79(A)
TGCCATGCCTTTCCACAGCTTTCTTGAACTTCTCTTGTGCCTCACTCACTTTCATTACT
GGAGAGATATGCAT**A**AT**C**A**A**TGGAAGCTTATAGGGAGAGGAGTAGCAAGAAGAGG
GTCAAGAAAGCTGTGGGAAGAAATGGCA
>O.punctata3_M74(A)_M77(C)_R79(G)
TGCCATGCCTTTCCACAGCTTTCTTGAACTTCTCTTGTGCCTCACTCACTTTCATTACT
GGAGAGATATGCAT**A**AT**C**A**G**TGGAAGCTTATAGGGAGAGGAGTAGCAAGAAGAGG
GTCAAGAAAGCTGTGGGAAGAAATGGCA
>O.punctata4_M74(A)_M77(A)_R79(G)
TGCCATGCCTTTCCACAGCTTTCTTGAACTTCTCTTGTGCCTCACTCACTTTCATTACT
GGAGAGATATGCAT**A**AT**A**A**G**TGGAAGCTTATAGGGAGAGGAGTAGCAAGAAGAGG
GTCAAGAAAGCTGTGGGAAGAAATGGCA
>O.punctata5_M74(C)_M77(A)_R79(G)
TGCCATGCCTTTCCACAGCTTTCTTGAACTTCTCTTGTGCCTCACTCACTTTCATTACT
GGAGAGATATGCAT**C**AT**A**A**G**TGGAAGCTTATAGGGAGAGGAGTAGCAAGAAGAGG
GTCAAGAAAGCTGTGGGAAGAAATGGCA
>O.punctata6_M74(C)_M77(A)_R79(A)
TGCCATGCCTTTCCACAGCTTTCTTGAACTTCTCTTGTGCCTCACTCACTTTCATTACT
GGAGAGATATGCAT**C**AT**A**A**A**TGGAAGCTTATAGGGAGAGGAGTAGCAAGAAGAGG
GTCAAGAAAGCTGTGGGAAGAAATGGCA
>O.minuta
TGCCATGCCTTTCCACAGCTTTCTTGAACTTCTCTTGTGCCTCACTCACTTTCATTACT
GGAGAGATATGCATCATCAATGGAAGCTTATAGGGAGAGGAT**AG**CAAGAAGAGGGT
CAAGAAAGCTGTGGGAAGAAATGGCA
GROUP 2:
>O.officinalis
TGCCATGCCTTTCCACAGCTTTCTTGAACTTCTCTTGTGCCTCACTCACTTTCATTACT
GGAGAGATATGCATCATCAGTGGAAGCTTATAGGGAGAGGGAGTACAAGAAGAGGG
TCAAGAAAGCTGTGGGAAGAAATGGCA
>O.alta_103(A)
TGCCATGCCTTTCCACAGCTTTCTTGAACTTCTCTTGTGCCTCACTCACTTTCATTACT
GGAGAGATATGCATCATCAGTGGAAGCTTATAGGGAGAGGAGT**A**CAAGAAGAGGGT
CAAGAAAGCTGTGGGAAGAAATGGCA
>O.glaberrima_103(G)
TGCCATGCCTTTCCACAGCTTTCTTGAACTTCTCTTGTGCCTCACTCACTTTCATTACT
GGAGAGATATGCATCATCAGTGGAAGCTTATAGGGAGAGGAGT**G**CAAGAAGAGGGT
CAAGAAAGCTGTGGGAAGAAATGGCA
>O.nivara_103(A)
TGCCATGCCTTTCCACAGCTTTCTTGAACTTCTCTTGTGCCTCACTCACTTTCATTACT
GGAGAGATATGCATCATCAGTGGAAGCTTATAGGGAGAGGAGT**A**CAAGAAGAGGGT
CAAGAAAGCTGTGGGAAGAAATGGCA
>O.rufipogon_103(A)

TGCCATGCCTTTCCACAGCTTTCTTGAACTTCTCTTGTGCCTCACTCACTTTCATTACT
GGAGAGATATGCATCATCAGTGGAAGCTTATAGGGAGAGGAGT**A**CAAGAAGAGGGT
CAAGAAAGCTGTGGGAAGAAATGGCA
>O.sativa(i)1_R103(A)
TGCCATGCCTTTCCACAGCTTTCTTGAACTTCTCTTGTGCCTCACTCACTTTCATTACT
GGAGAGATATGCATCATCAGTGGAAGCTTATAGGGAGAGGAGT**A**CAAGAAGAGGGT
CAAGAAAGCTGTGGGAAGAAATGGCA
>O.sativa(i)2_R103(G)
TGCCATGCCTTTCCACAGCTTTCTTGAACTTCTCTTGTGCCTCACTCACTTTCATTACT
GGAGAGATATGCATCATCAGTGGAAGCTTATAGGGAGAGGAGT**G**CAAGAAGAGGGT
CAAGAAAGCTGTGGGAAGAAATGGCA
>O.barthii1_R103(A)
TGCCATGCCTTTCCACAGCTTTCTTGAACTTCTCTTGTGCCTCACTCACTTTCATTACT
GGAGAGATATGCATCATCAGTGGAAGCTTATAGGGAGAGGAGT**A**CAAGAAGAGGGT
CAAGAAAGCTGTGGGAAGAAATGGCA
>O.barthii2_R103(G)
TGCCATGCCTTTCCACAGCTTTCTTGAACTTCTCTTGTGCCTCACTCACTTTCATTACT
GGAGAGATATGCATCATCAGTGGAAGCTTATAGGGAGAGGAGT**G**CAAGAAGAGGGT
CAAGAAAGCTGTGGGAAGAAATGGCA
>O.australiensis1_R103(A)
TGCCATGCCTTTCCACAGCTTTCTTGAACTTCTCTTGTGCCTCACTCACTTTCATTACT
GGAGAGATATGCATCATCAGTGGAAGCTTATAGGGAGAGGAGT**A**CAAGAAGAGGGT
CAAGAAAGCTGTGGGAAGAAATGGCA
>O.australiensis2_R103(G)
TGCCATGCCTTTCCACAGCTTTCTTGAACTTCTCTTGTGCCTCACTCACTTTCATTACT
GGAGAGATATGCATCATCAGTGGAAGCTTATAGGGAGAGGAGT**G**CAAGAAGAGGGT
CAAGAAAGCTGTGGGAAGAAATGGCA
>O.sativa(j)1_R103(A)
TGCCATGCCTTTCCACAGCTTTCTTGAACTTCTCTTGTGCCTCACTCACTTTCATTACT
GGAGAGATATGCATCATCAGTGGAAGCTTATAGGGAGAGGAGT**A**CAAGAAGAGGGT
CAAGAAAGCTGTGGGAAGAAATGGCA
>O.sativa(j)2_R103(G)
TGCCATGCCTTTCCACAGCTTTCTTGAACTTCTCTTGTGCCTCACTCACTTTCATTACT
GGAGAGATATGCATCATCAGTGGAAGCTTATAGGGAGAGGAGT**G**CAAGAAGAGGGT
CAAGAAAGCTGTGGGAAGAAATGGCA
GROUP 3:
>O.coarctata
TGCCATTTCTTCCCACAGCTTTCTTGACCCTCTTCTTGCTCTCTCCCTGTAAGCTTCCA
TT

```
O.sativa                  TGCCATGCCTTTCCACAGCTTTCTTGAACTTCTCTTGTGCCTCACTCACTTTCATTACTG 60
O.coarctata               TGCCATTTCTTCCCACAGCTTTCTTGACCCTCTTCTTGCTCTCTCCCTGTAAGCTTCCAT 60
O.coarctataTKM-2016       TGCCATTTCTTCCCACAGCTTTCTTGACCCTCTTCTTGCTCTCTCCCTGTAAGCTTCCAT 60
                          ******  *** *************** * ***  *    *** * *  *    ** *

O.sativa                  GAGAGATATGCATCATCAGTGGAAGCTTATAGGGAGAGGAGTACAAGAAGAGGGTCAAGA 120
O.coarctata               TGATGATGTTTCTCCAGTGATG--------AAATGAATGAGGCACAAGAGAAGTTCAAGA 112
O.coarctataTKM-2016       TGATGATGTTTCTCCAGTGATG--------AAATGAATGAGGCACAAGAGAAGTTCAAGA 112
                              *** *   **    *  *        *     * ***    *  *** * ******

O.sativa                  AAGCTGTGGGAAGAAATGGCA  141
O.coarctata               AAGCTGTGGAAAGGAATGGCA  133
O.coarctataTKM-2016       AAGCTGTGGAAAGGAATGGCA  133
                          ********* *** *******
```

Supplementary Figure 2: Pre-*miR396c* sequence from halophytic wild rice *Oryza coarctata* is significantly different from O. sativa pre-*mir396c*. *O. coarctata* pre-miR sequence reported here (marked *O. coarctata*) is identical to that reported in the *O. coarctata* genome (marked *O. coarctata* TKM-2016; Accession no: WLYV01055019.1: (region 44700-44832 bp). Mature miR and miR* region are highlighted in red and yellow respectively and bases differing in these regions in *O. coarctata* are highlighted in grey.

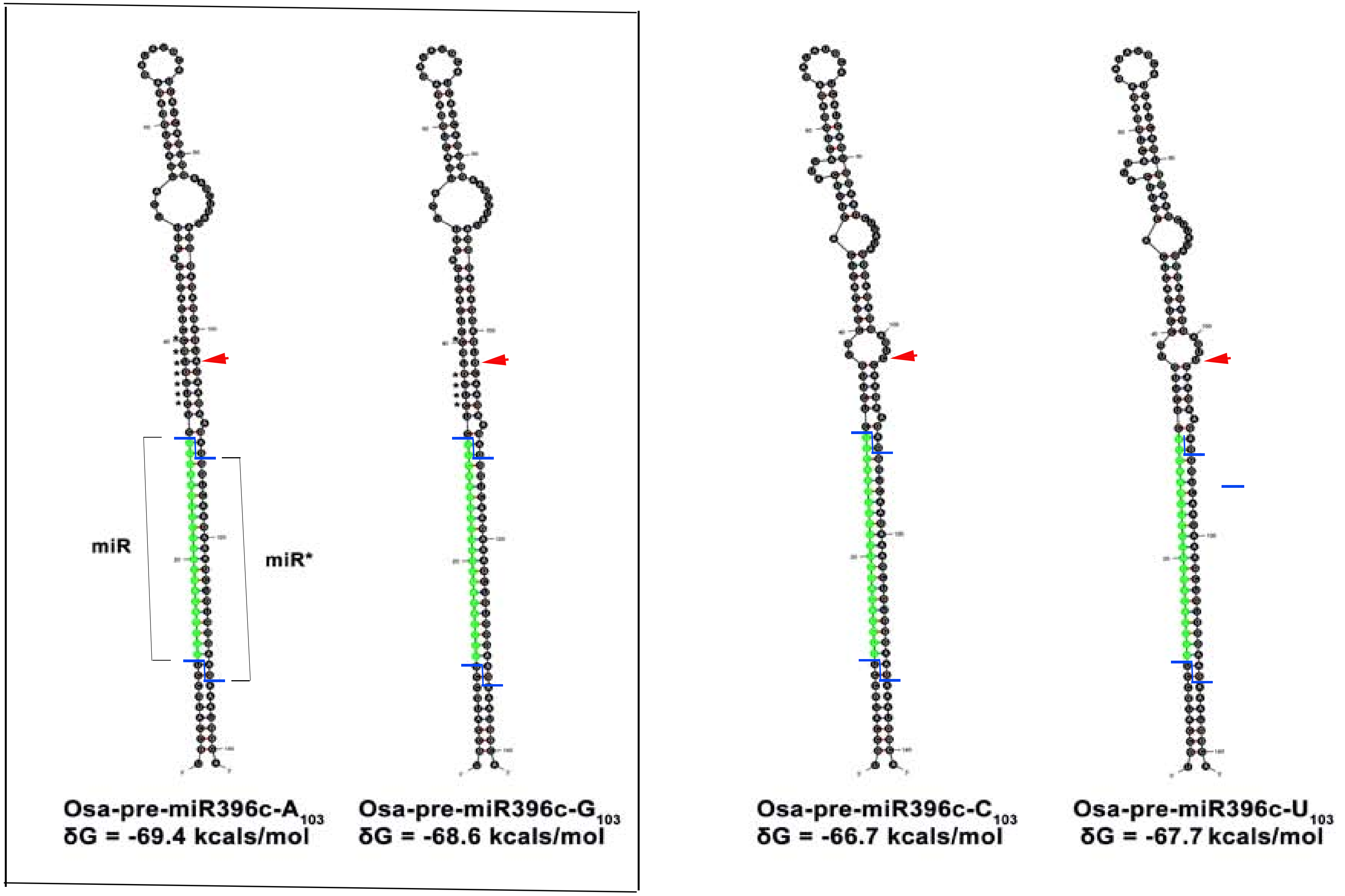

Supplementary Figure 3: Secondary structures of Osa-pre-miR396c sequences with either A-, G-, C- or U- at the 103rd position (red arrow) in the *O. sativa*, as predicted using Mfold. Pre-miRNA sequence results in a perfect U_{38}-A_{103} base pair while the presence of a 'G' at the 103rd position disrupts this base pairing, resulting in a minor increase in the $\Delta G_{folding}$ of the pre-miR396c by -0.8 kcals/mol. In addition, the presence of A_{103} results in a contiguous stretch of seven base paired residues (marked by *), just above a five bp bulge in the upper region, in contrast to the presence of G_{103}, resulting in 5 base paired residues flanking U_{38}-G_{103} and G_{39}-U_{102}. Pre-miR396c-A/G_{103} have been reported (boxed), while pre-miR396c-C/U_{103} have not been reported to occur naturally. miR396c (marked miR; in green) and miR396c* (miR*) are indicated. Blue lines indicate cleavge sites in pre-mir396c.

```
CT856578.1      ------------------------------------------------------------ 0
CT829989.1      ------------------------------------------------------------ 0
CK053360.1      ------------------------------------------------------------ 0
CK045291.1      ------------------------------------------------------------ 0
AK062523.1      CTTTTTAACCCATCCAATGCCCCTTGTTGCTGCAACCTCATTTAGATCTCATCCTTCTCT 60
CI029080.1      ------------------------------------------------------------ 0

CT856578.1      ------------------------------------------------------------ 0
CT829989.1      ---------------------------------GTACGTCTACCACAGCTTGCAGCCATA 27
CK053360.1      ------------------------------------------------------------ 0
CK045291.1      ------------------------------------------------------------ 0
AK062523.1      TCATATATACATGCATACTGCTATAAGTCTACTGTACGTCTACCACAGCTTGCAGCCATA 120
CI029080.1      ------------------------------------------------------------ 0

CT856578.1      ------------------------------------------------------------ 0
CT829989.1      AAGCTCTTCGTCCTCTCTCTTTCTACGGCTGTGGAGCTGAAGCATGGCCTAGCTGAGCTA 87
CK053360.1      ------------CTCTCTCTTTCTACGGCTGTGGAGCTGAAGCATGGCCTAGCTGAGCTA 48
CK045291.1      ------------------------------------------------------------ 0
AK062523.1      AAGCTCTTCGTCCTCTCTCTTTCTACGGCTGTGGAGCTGAAGCATGGCCTAGCTGAGCTA 180
CI029080.1      ------------------------ACGGCTGTGGAGCTGAAGCATGGCCTAGCTGAGCTA 36

CT856578.1      ---------------------------------------------AGACGACAGATGGCA 15
CT829989.1      GCAGCTGGTTCTTGGTTATATAAAGAGAGATAGCTAGGATCAAGAGAGAGGCAGATGGCA 147
CK053360.1      GCAGCTGGTTCTTGGTTATATAAAGAGAGATAGCTAGGATCAAGAGAGAGGCAGATGGCA 108
CK045291.1      -----------------------------------------------CGGCACGAGGGCA 13
AK062523.1      GCAGCTGGTTCTTGGTTATATAAAGAGAGATAGCTAGGATCAAGAGAGAGGCAGATGGCA 240
CI029080.1      GCAGCTGGTTCTTGGTTATATAAAGAGAGATAGCTAGGATCAAGAGAGAGGCAGATGGCA 96
                                                 *   ** ****

CT856578.1      TGGAGAGGTGTTGCAATGTGCATTGGATGTGTAGATAGAACCTGCAGATCTCGATCGATC 75
CT829989.1      TGGAGAGGTGTTGCAATGTGCATTGGATGTGTAGATAGAACCTGCAGATCTCGATCGATC 207
CK053360.1      TGGAGAGGTGTTGCAATGTGCATTGGATGTGTAGATAGAGCCTGCAGATCTCGATCGATC 168
CK045291.1      TGGAGAGGTGTTGCAATGTGCATTGGATGTGTAGATAGAGCCTGCAGATCTCGATCGATC 73
AK062523.1      TGGAGAGGTGTTGCAATGTGCATTGGATGTGTAGATAGAGCCTGCAGATCTCGATCGATC 300
CI029080.1      TGGAGAGGTGTTGCAATGTGCATTGGATGTGTAGATAGAGCCTGCAGATCTCGATCGATC 156
                *************************************** ********************

CT856578.1      TCTTCAAGTCCATGCCATGCCTTTCCACAGCTTTCTTGAACTTCTCTTGTGCCTCACTCA 135
CT829989.1      TCTTCAAGTCCATGCCATGCCTTTCCACAGCTTTCTTGAACTTCTCTTGTGCCTCACTCA 267
CK053360.1      TCTTCAAGTCCATGCCATGCCTTTCCACAGCTTTCTTGAACTTCTCTTGTGCCTCACTCA 228
CK045291.1      TCTTCAAGTCCATGCCATGCCTTTCCACAGCTTTCTTGAACTTCTCTTGTGCCTCACTCA 133
AK062523.1      TCTTCAAGTCCATGCCATGCCTTTCCACAGCTTTCTTGAACTTCTCTTGTGCCTCACTCA 360
CI029080.1      TCTTCAAGTCCATGCCATGCCTTTCCACAGCTTTCTTGAACTTCTCTTGTGCCTCACTCA 216
                ************************************************************

CT850193.1      CTTTCATTACTGGAGAGATATGCATCATCAGTGGAAGCTTATAGGGAGATTAGTACAAGA 327
CT856578.1      CTTTCATTACTGGAGAGATATGCATCATCAGTGGAAGCTTATAGGGAGAGGAGTACAAGA 195
CT829989.1      CTTTCATTACTGGAGAGATATGCATCATCAGTGGAAGCTTATAGGGAGAGGAGTACAAGA 327
CK053360.1      CTTTCATTACTGGAGAGATATGCATCATCAGTGGAAGCTTATAGGGAGAGGAGTGCAAGA 288
CK045291.1      CTTTCATTACTGGAGAGATATGCATCATCAGTGGAAGCTTATAGGGAGAGGAGTGCAAGA 193
AK062523.1      CTTTCATTACTGGAGAGATATGCATCATCAGTGGAAGCTTATAGGGAGAGGAGTGCAAGA 420
CI029080.1      CTTTCATTACTGGAGAGATATGCATCATCAGTGGAAGCTTATAGGGAGAGGAGTGCAAGA 276
                *************************************************  *** *****

CT856578.1      AGAGGGTCAAGAAAGCTGTGGGAAGAAATGGCATTACAAGGACTTTGAATTCAGTTAGCA 255
CT829989.1      AGAGGGTCAAGAAAGCTGTGGGAAGAAATGGCATTACAAGGACTTTGAATTCAGTTAGCA 387
CK053360.1      AGAGGGTCAAGAAAGCTGTGGGAAGAAATGGCATTACAAGGACTTTGAATTCAGTTAGCA 348
CK045291.1      AGAGGGTCAAGAAAGCTGTGGGAAGAAATGGCATTACAAGGACTTTGAATTCAGTTAGCA 253
AK062523.1      AGAGGGTCAAGAAAGCTGTGGGAAGAAATGGCATTACAAGGACTTTGAATTCAGTTAGCA 480
CI029080.1      AGAGGGTCAAGAAAGCTGTGGGAAGAAATGGCATTACAAGGACTTTGAATTCAGTTAGCA 336
                ************************************** **     *      **
```

```
CT856578.1      ATGCCATGAGACAGGCAGAGAGAGAGAGAGAGAGAGAGATGT----------------GA 299
CT829989.1      ATGCCATGAGACAGGCAGAGAGAGAGAGAGAGAGAGAGATGT----------------GA 431
CK053360.1      ATGCCATGAGACAGGCAGAGAGAGAGAGAG--------------AGAGAGAGAGATGTGA 394
CK045291.1      ATGCCATGAGACAGGCAGAGAGAGAGAGAG--------------AGAGAGAGAGATGTGA 299
AK062523.1      ATGCCATGAGACAGGCAGAGAGAGAGAGAGAGAGAGAGAGAGAGAGAGAGAGAGATGTGA 540
CI029080.1      ATGCCATGAGACAGGCAGAGAGAGAGAGAGAGAGAGAGAGAGAGAGAGAGAGAGATGTGA 396
                *   *             *** ********                            **

CT856578.1      TGCTAATTTTCCATTTCATGTAAGTTAGCTTTTCATAATTAGATCAAGTTTTCTCTACTT 359
CT829989.1      TGCTAATTTTCCATTTCATTGACACTGGATGTGCGATTGGATCTATCCTCTTTGTAGGGA 491
CK053360.1      TGCTAATTTTCCATTTCATGTAAGTTAGCTTTTCATAATTAGATCAAGTTTTCT------ 448
CK045291.1      TGCTAATTTTCCATTTC------------------------------------------- 316
AK062523.1      TGCTAATTTTCCATTTCATGTAAGTTAGCTTTTCATAATTAGATCAAGTTTTCT------ 594
CI029080.1      TGCTAATTTTCCATTTCATGTAAGTTAGCTTTTCATATAGATCCAAGTTTTCTC------ 450
                 ****** *  *  *

CT856578.1      AATTTGGTGCCCTTTTTGCTTACGTTTTTTTTTCGCTGGGAGGTTTTGCTTACTTAATTT 419
CT829989.1      GGCATTTGA--------AGGCACTTCTC--ACATGTTCAAGGAATGTTCTTTCATCTCTG 541
CK053360.1      -------------------CTACTTAAA--AAAAAAAAAAAAAAAAA------------- 474
CK045291.1      ------------------------------------------------------------ 316
AK062523.1      -------------------CT--------------------------------------- 596
CI029080.1      -------------------TAAA------------------------------------- 454

CT856578.1      AAGTGCCTGCTAGTTTCTTTTTGCTAGTCCAGCTAGCCACTGCATATTTAA---ATTTTA 476
CT829989.1      AAGTGTGGAACAAAATCTGTATTTGGAAGGGGTTAGGTCACAACTCTT------------ 589
CK053360.1      ------------------------------------------------------------ 474
CK045291.1      ------------------------------------------------------------ 316
AK062523.1      ------------------------------------------------------------ 596
CI029080.1      ------------------------------------------------------------ 454

CT856578.1      TATCTGCCACTGTCAGCTCCAATTA--TTTGTCGTTTGTTTTTTCAAACAACAGTCTGCA 534
CT829989.1      TGCCTAACAACTTGGATTTGAAAAG--CTGGTGGTGCTCTCTTTCAGCCATT------CA 641
CK053360.1      ------------------------------------------------------------ 474
CK045291.1      ------------------------------------------------------------ 316
AK062523.1      ------------------------------------------------------------ 596
CI029080.1      ------------------------------------------------------------ 454

CT856578.1      TGCATTTCGGCATCAATAGAGGACCATCACTGTGTAGCT-CGATCATTGTAGCTCATGAA 593
CT829989.1      ACCAAAACCCCAGCAAAAAAGAATTATGGGTTGTTTGATCACTTCCTGGTAGAACATCTG 701
CK053360.1      ------------------------------------------------------------ 474
CK045291.1      ------------------------------------------------------------ 316
AK062523.1      ------------------------------------------------------------ 596
CI029080.1      ------------------------------------------------------------ 454

CT856578.1      ATTGATGTCTCTTCCGTAAAGGCGTCTTCACT-TTTTTCTCTTACCATGCATATCATGCA 652
CT829989.1      GTTGGAG---------------CGGAATAA------------------GCGTATATTCCA 728
CK053360.1      ------------------------------------------------------------ 474
CK045291.1      ------------------------------------------------------------ 316
AK062523.1      ------------------------------------------------------------ 596
CI029080.1      ------------------------------------------------------------ 454

CT856578.1      TCAAGGCTAAAAAGGAATTCAACCTTTTCCATTTTGCTTTGCTTAGTACTACAGTACCAT 712
CT829989.1      --------AAAAACTTTGTCATCGGCTGATAGGGTGGCTTCCTTGATTCTGCAAGATCTT 780
CK053360.1      ------------------------------------------------------------ 474
CK045291.1      ------------------------------------------------------------ 316
AK062523.1      ------------------------------------------------------------ 596
CI029080.1      ------------------------------------------------------------ 454

CT856578.1      GTAGGGCTTCTCGCA----TGCATG-ACAAGAAATGCAGCAAGATT---GTGGTGTT--G 762
CT829989.1      GACCTGAGACATTCAGCTTTCCAATCTCAGTGAGGCCGTCCCTCTTTTTGTAATTTT--T 838
CK053360.1      ------------------------------------------------------------ 474
CK045291.1      ------------------------------------------------------------ 316
```

```
AK062523.1      ------------------------------------------------------------ 596
CI029080.1      ------------------------------------------------------------ 454

CT856578.1      AGTTTGA---ATACTCAATGGGAGCAGTATATAACCAATCTTGATGAATTTATCATCACG 819
CT829989.1      TGTTTTTTTCGCTTTGCTTTTGTGCTCTGTTGAGGTAAACAACTTTTTCTTCTTCTAATA 898
CK053360.1      ------------------------------------------------------------ 474
CK045291.1      ------------------------------------------------------------ 316
AK062523.1      ------------------------------------------------------------ 596
CI029080.1      ------------------------------------------------------------ 454

CT856578.1      GAATGGGTCTATGTTTTTTCTTTTTTTGAATAATCAATGGTTAAGTGCATTCAGATTTTT 879
CT829989.1      TATTCCTTCTAACGTTCTGCGTCGTTTCCCTAAAAAAAAAAAAAG--------------- 943
CK053360.1      ------------------------------------------------------------ 474
CK045291.1      ------------------------------------------------------------ 316
AK062523.1      ------------------------------------------------------------ 596
CI029080.1      ------------------------------------------------------------ 454

CT856578.1      CACATTTTTTAAATTAAGTCCAAGGCTAATTTTGGGGATAATATATTATATATATATATT 939
CT829989.1      ------------------------------------------------------------ 943
CK053360.1      ------------------------------------------------------------ 474
CK045291.1      ------------------------------------------------------------ 316
AK062523.1      ------------------------------------------------------------ 596
CI029080.1      ------------------------------------------------------------ 454

CT856578.1      TTATATTTTTTTGTACGCGGGCTCGATAGTGAGGATGTAAAAACGTATAAAAACATTCTC 999
CT829989.1      ------------------------------------------------------------ 943
CK053360.1      ------------------------------------------------------------ 474
CK045291.1      ------------------------------------------------------------ 316
AK062523.1      ------------------------------------------------------------ 596
CI029080.1      ------------------------------------------------------------ 454

CT856578.1      CATCTCATATTCAAACGATCGAAAGGGAGGAA-AAAAA----------AATAG------- 1041
CT829989.1      ------------------------------------------------------------ 943
CK053360.1      ------------------------------------------------------------ 474
CK045291.1      ------------------------------------------------------------ 316
AK062523.1      ------------------------------------------------------------ 596
CI029080.1      ------------------------------------------------------------ 454

CT856578.1      ----------------GATTAATGAGAGGAGGGGGGGCAGCTTCCACAGCCCGCCACAAC 1085
CT829989.1      ------------------------------------------------------------ 943
CK053360.1      ------------------------------------------------------------ 474
CK045291.1      ------------------------------------------------------------ 316
AK062523.1      ------------------------------------------------------------ 596
CI029080.1      ------------------------------------------------------------ 454

CT856578.1      CACACCCCACCCCCAACACGAACTGGGGCTCGAGGAATAAGAAATAAAAATTGAAAAATA 1145
CT829989.1      ------------------------------------------------------------ 943
CK053360.1      ------------------------------------------------------------ 474
CK045291.1      ------------------------------------------------------------ 316
AK062523.1      ------------------------------------------------------------ 596
CI029080.1      ------------------------------------------------------------ 454

CT856578.1      TAAAAAAATTAAAAAAATAATGAAGAGCATCTAGTTGCTGGACGCTGGGGTCTGCGTGCA 1205
CT829989.1      ------------------------------------------------------------ 943
CK053360.1      ------------------------------------------------------------ 474
CK045291.1      ------------------------------------------------------------ 316
AK062523.1      ------------------------------------------------------------ 596
CI029080.1      ------------------------------------------------------------ 454
```

```
CT856578.1      CGCTCCACTGCGGCTGCCCC      1225
CT829989.1      --------------------      943
CK053360.1      --------------------      474
CK045291.1      --------------------      316
AK062523.1      --------------------      596
CI029080.1      --------------------      454
```

Supplementary Figure 4: Selected cDNA/ESTs corresponding to the *O. sativa* Pri-*miR396c* locus (Os02g0804000) aligned using Clustal Omega. Of the ESTs, only CT829989.1 shows correct splicing (5' splice junction highlighted in grey; correctly spliced 3' end in pink) while the remaining ESTs show partial intron retention (indicated in red). The *Osa-pre-miR396c* sequence is highlighted in yellow, mature Osa-*miR396c* and mir396c* regions in blue and dark green respectively while the SNP *rs10234287911* is highlighted in fluorescent green.

```
Mundon-2        GAGAGATATGCATCATCAGTGGAAGCTTATAGGGAGAGGAGTGCAAGAAGAGGGTCAAGA 120
Gheus           GAGAGATATGCATCATCAGTGGAAGCTTATAGGGAGAGGAGTGCAAGAAGAGGGTCAAGA 120
Pokkali-4       GAGAGATATGCATCATCAGTGGAAGCTTATAGGGAGAGGAGTGCAAGAAGAGGGTCAAGA 120
Altaluti        GAGAGATATGCATCATCAGTGGAAGCTTATAGGGAGAGGAGTGCAAGAAGAGGGTCAAGA 120
Katrangi        GAGAGATATGCATCATCAGTGGAAGCTTATAGGGAGAGGAGTGCAAGAAGAGGGTCAAGA 120
Darsal          GAGAGATATGCATCATCAGTGGAAGCTTATAGGGAGAGGAGTGCAAGAAGAGGGTCAAGA 120
Marisal         GAGAGATATGCATCATCAGTGGAAGCTTATAGGGAGAGGAGTGCAAGAAGAGGGTCAAGA 120
Nonasoren       GAGAGATATGCATCATCAGTGGAAGCTTATAGGGAGAGGAGTGCAAGAAGAGGGTCAAGA 120
Kalanuia        GAGAGATATGCATCATCAGTGGAAGCTTATAGGGAGAGGAGTGCAAGAAGAGGGTCAAGA 120
Patnai-23       GAGAGATATGCATCATCAGTGGAAGCTTATAGGGAGAGGAGTGCAAGAAGAGGGTCAAGA 120
Mundon-1        GAGAGATATGCATCATCAGTGGAAGCTTATAGGGAGAGGAGTGCAAGAAGAGGGTCAAGA 120
Hamilton        GAGAGATATGCATCATCAGTGGAAGCTTATAGGGAGAGGAGTGCAAGAAGAGGGTCAAGA 120
Pallipuram      GAGAGATATGCATCATCAGTGGAAGCTTATAGGGAGAGGAGTGCAAGAAGAGGGTCAAGA 120
Kagga           GAGAGATATGCATCATCAGTGGAAGCTTATAGGGAGAGGAGTGCAAGAAGAGGGTCAAGA 120
Matla-1         GAGAGATATGCATCATCAGTGGAAGCTTATAGGGAGAGGAGTGCAAGAAGAGGGTCAAGA 120
Matla-2         GAGAGATATGCATCATCAGTGGAAGCTTATAGGGAGAGGAGTGCAAGAAGAGGGTCAAGA 120
Pokkali-2       GAGAGATATGCATCATCAGTGGAAGCTTATAGGGAGAGGAGTGCAAGAAGAGGGTCAAGA 120
Pokkali-3       GAGAGATATGCATCATCAGTGGAAGCTTATAGGGAGAGGAGTGCAAGAAGAGGGTCAAGA 120
FL478           GAGAGATATGCATCATCAGTGGAAGCTTATAGGGAGAGGAGTGCAAGAAGAGGGTCAAGA 120
IR28            GAGAGATATGCATCATCAGTGGAAGCTTATAGGGAGAGGAGTGCAAGAAGAGGGTCAAGA 120
IR29            GAGAGATATGCATCATCAGTGGAAGCTTATAGGGAGAGGAGTGCAAGAAGAGGGTCAAGA 120

Chettvirrpu     GAGAGATATGCATCATCAGTGGAAGCTTATAGGGAGAGGAGTACAAGAAGAGGGTCAAGA 120
Nonabokra-1     GAGAGATATGCATCATCAGTGGAAGCTTATAGGGAGAGGAGTACAAGAAGAGGGTCAAGA 120
Nonabokra-2     GAGAGATATGCATCATCAGTGGAAGCTTATAGGGAGAGGAGTACAAGAAGAGGGTCAAGA 120
Nonabokra-3     GAGAGATATGCATCATCAGTGGAAGCTTATAGGGAGAGGAGTACAAGAAGAGGGTCAAGA 120
NonabokraI      GAGAGATATGCATCATCAGTGGAAGCTTATAGGGAGAGGAGTACAAGAAGAGGGTCAAGA 120
Hoogla          GAGAGATATGCATCATCAGTGGAAGCTTATAGGGAGAGGAGTACAAGAAGAGGGTCAAGA 120
Rupsal          GAGAGATATGCATCATCAGTGGAAGCTTATAGGGAGAGGAGTACAAGAAGAGGGTCAAGA 120
Kaksal          GAGAGATATGCATCATCAGTGGAAGCTTATAGGGAGAGGAGTACAAGAAGAGGGTCAAGA 120
Jingasal        GAGAGATATGCATCATCAGTGGAAGCTTATAGGGAGAGGAGTACAAGAAGAGGGTCAAGA 120
Kamini          GAGAGATATGCATCATCAGTGGAAGCTTATAGGGAGAGGAGTACAAGAAGAGGGTCAAGA 120
Anakodan        GAGAGATATGCATCATCAGTGGAAGCTTATAGGGAGAGGAGTACAAGAAGAGGGTCAAGA 120
Orumundakon1    GAGAGATATGCATCATCAGTGGAAGCTTATAGGGAGAGGAGTACAAGAAGAGGGTCAAGA 120
Orumundakon2    GAGAGATATGCATCATCAGTGGAAGCTTATAGGGAGAGGAGTACAAGAAGAGGGTCAAGA 120
Korgil          GAGAGATATGCATCATCAGTGGAAGCTTATAGGGAGAGGAGTACAAGAAGAGGGTCAAGA 120
Kalamocha       GAGAGATATGCATCATCAGTGGAAGCTTATAGGGAGAGGAGTACAAGAAGAGGGTCAAGA 120
Aduisen-1       GAGAGATATGCATCATCAGTGGAAGCTTATAGGGAGAGGAGTACAAGAAGAGGGTCAAGA 120
Aduisen-2       GAGAGATATGCATCATCAGTGGAAGCTTATAGGGAGAGGAGTACAAGAAGAGGGTCAAGA 120
Kaatuponni      GAGAGATATGCATCATCAGTGGAAGCTTATAGGGAGAGGAGTACAAGAAGAGGGTCAAGA 120
Talmugur-1      GAGAGATATGCATCATCAGTGGAAGCTTATAGGGAGAGGAGTACAAGAAGAGGGTCAAGA 120
Talmugur-2      GAGAGATATGCATCATCAGTGGAAGCTTATAGGGAGAGGAGTACAAGAAGAGGGTCAAGA 120
Talmugur-3      GAGAGATATGCATCATCAGTGGAAGCTTATAGGGAGAGGAGTACAAGAAGAGGGTCAAGA 120
Dudheswar       GAGAGATATGCATCATCAGTGGAAGCTTATAGGGAGAGGAGTACAAGAAGAGGGTCAAGA 119
```

Supplementary Figure 5: Validation and distribution of SNP- rs34287911 (G/A) alleles among 43 Oryza landraces. A 141 bp fragment of pre-*miRNA396c* was amplified from 43 landraces. Among 43 landraces analyzed, 22 showed the presence of an ‘A’ base at the 103rd position whereas 21 landraces had a ‘G’ at the same position. Only bases 61-108 are shown for ease of alignment.

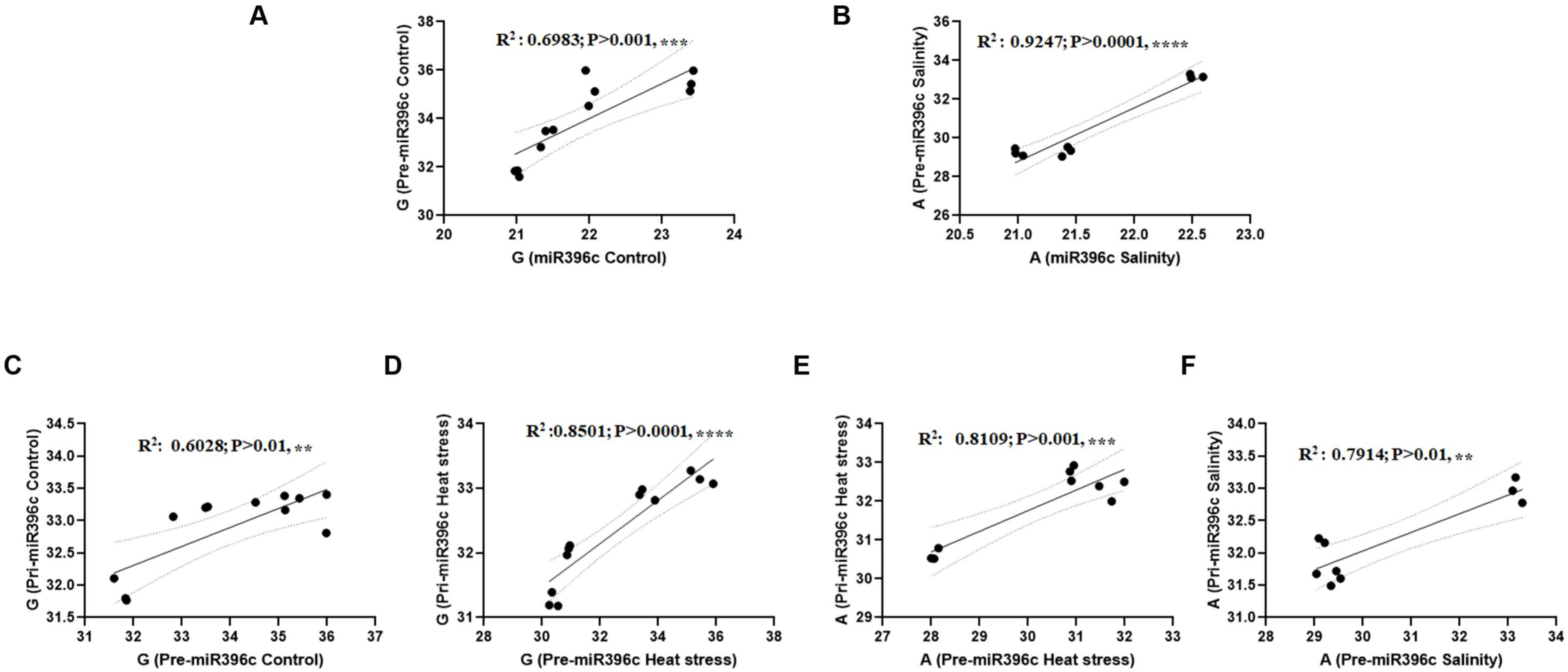

Supplementary Figure 6: Correlation analysis between (a) Pre-miR396c /mature miR396c expression under control conditions in rice landraces grouped ‘G’ and (B) pre-miR396c/mature miR396c expression under salinity in rice landraces grouped ‘A’ , (C) Pri-miR396c /pre-miR396c expression under control conditions in rice landraces grouped ‘G’, (D) Pri-miR396c /pre-miR396c expression under heat stress in rice landraces grouped ‘G’; (E) Pri-miR396c /pre-miR396c expression under heat stress in rice landraces grouped ‘A’ and (F) Pri-miR396c /pre-miR396c expression under salinity in rice landraces grouped ‘A’. R^2 values and probability are indicated.

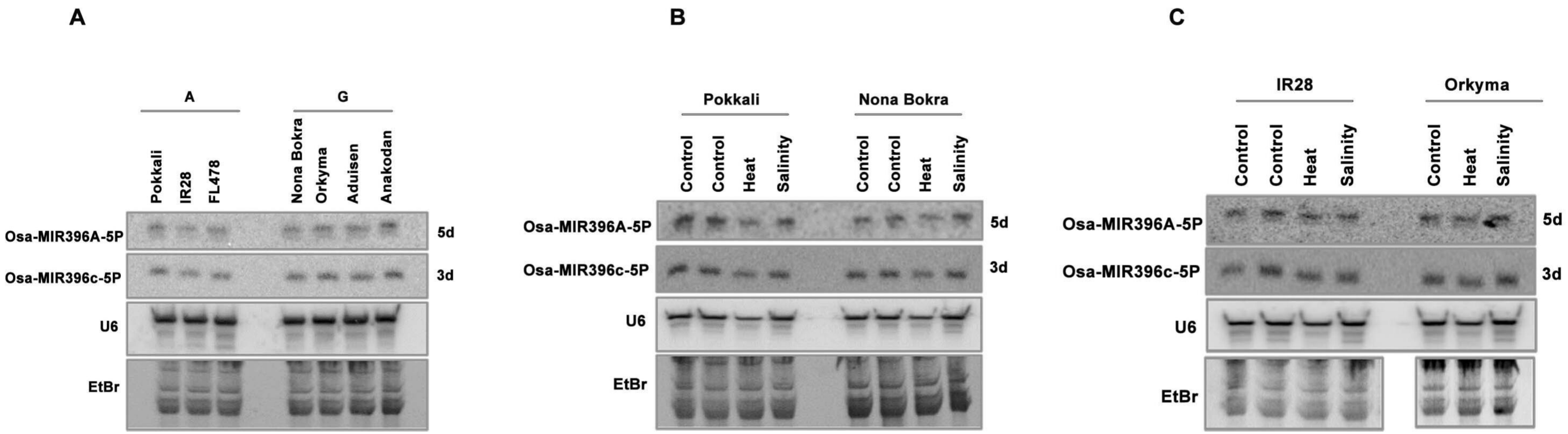

Supplementary Figure 7: Small RNA Northern blot analysis of *miR396a* and *mIR396c* expression in leaf tissues of *O. sativa.* A. *miR396a* and *miR396c* expression in control (untreated) leaves of rice landraces grouped 'A' [Pokkali, IR28, FL478] or 'G' [Nona Bokra, Orkyma, Aduisen, Anakodan]. B. *miR396a* and *miR396c* expression in leaves of Pokkali and Nonabokra landraces under control (untreated), heat and salinity. C. *miR396a* and *miR396c* expression in leaves of IR28 and Orkyma landraces under control (untreated), heat and salinity. In each case blots were stripped and re-probed with U6 for loading control. Ehidium bromide (EtBr) stained PAGE gels are also shown. 3d and 5d indicated 3 and 5 day auto-radiographic exposure times respectively.

Northern blots as well as ethidium bromide stained gels shown in Supplementary Fig 7 A, B and C are shown in Supplementary Figure 12 as un-cropped images for reference.

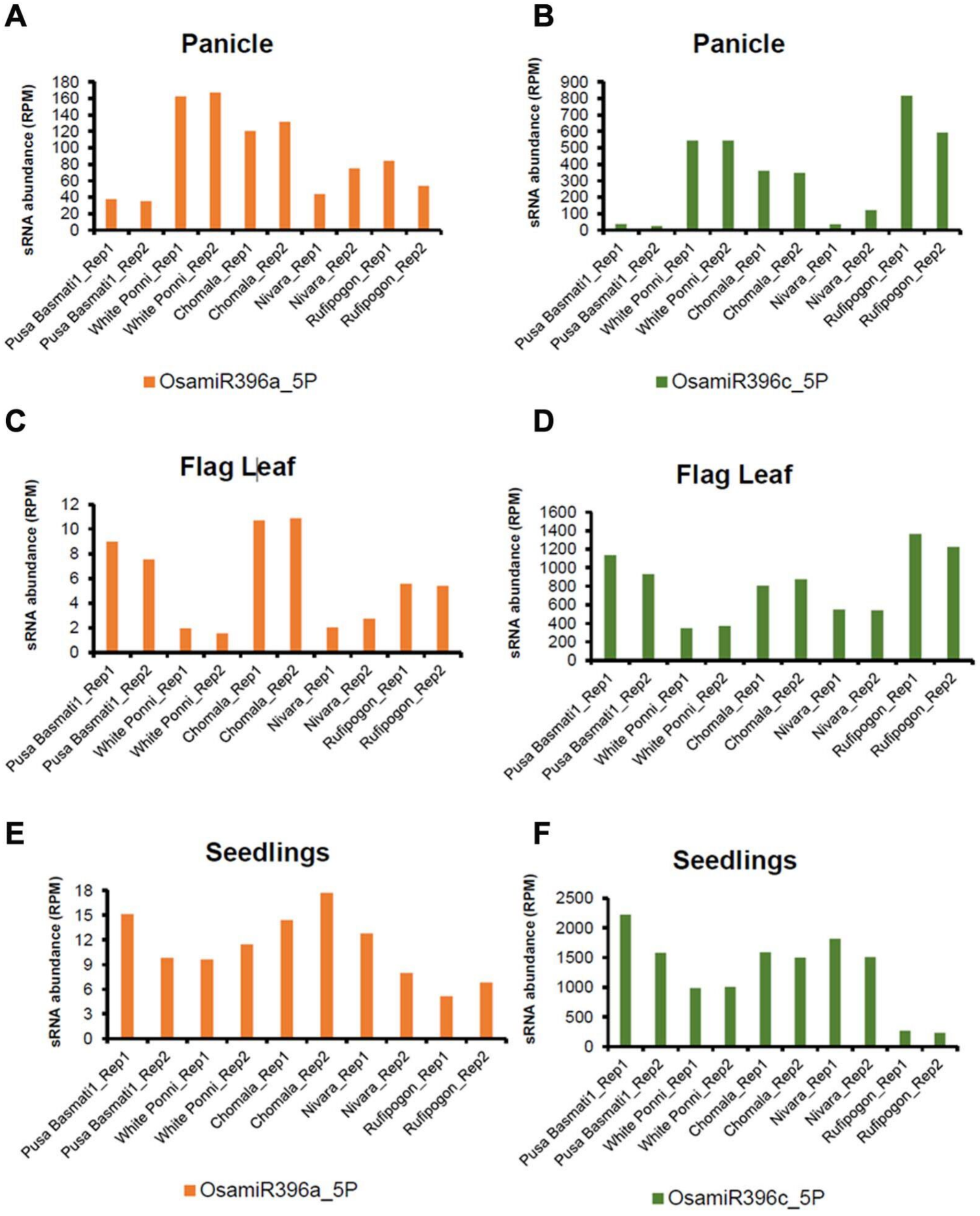

Supplementary Figure 8: Small RNA sequencing (NGS) data from Chenna et al., (2019) showing *miR396a-5p* (A, C, E) or *miR396c-5p* (B, D, F) abundance (RPM) in two replicates (tissues: panicle, flag leaf and seedling) each for rice landraces (Pusa Basmati1, White Ponni, Chomala) or wild rice species (*O. nivara, O. rufipogon*).

A

B

C

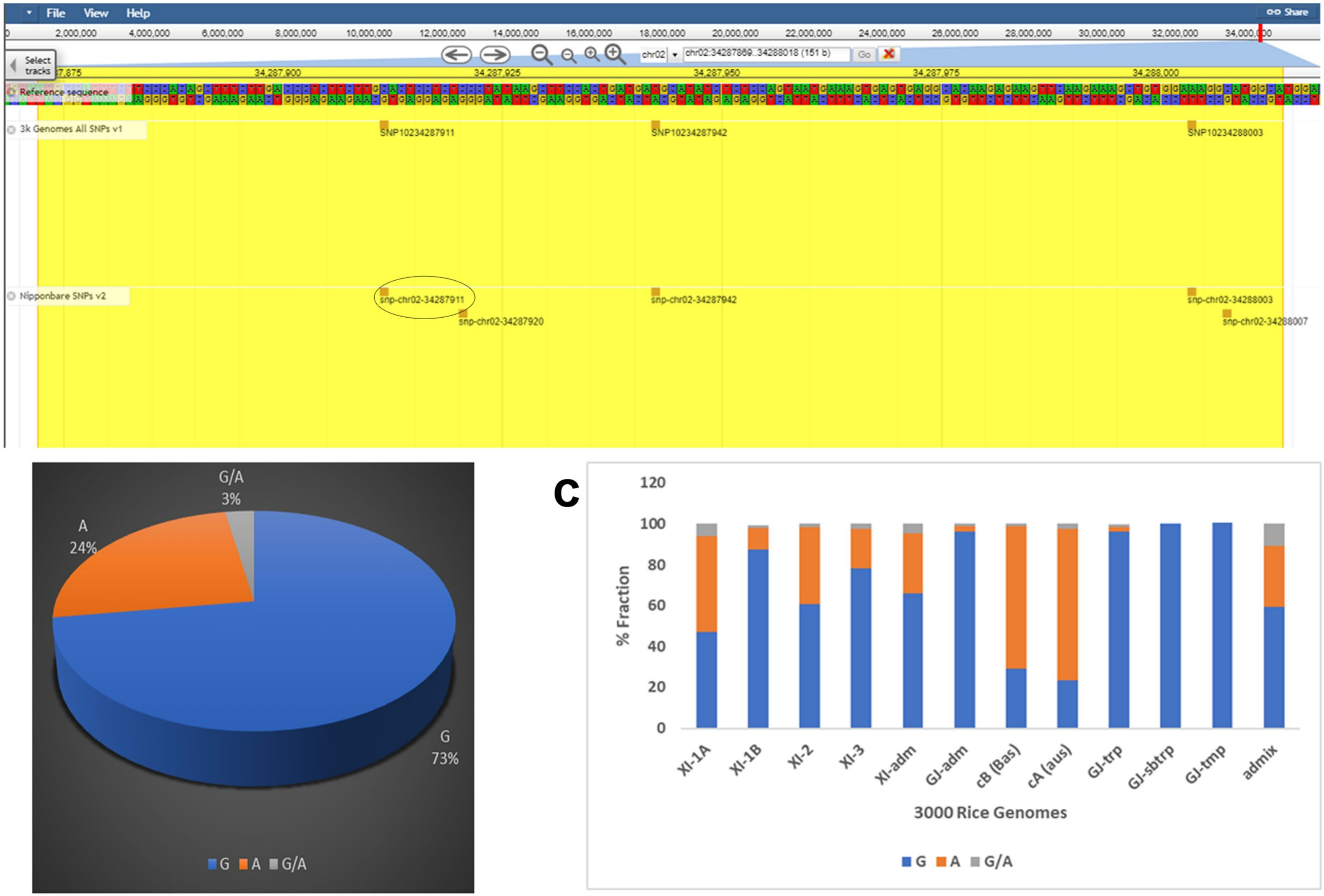

Supplementary Figure 9: A: SNP rs10234287911 in 3000 rice genome accessions (circled). B. Pie chart representation proportion of 3000 rice genome accessions with SNP rs34287911 (G, A or G/A). C: Distribution of SNP-rs34287911 (G/A) alleles among sub-populations of 3010 *O. sativa* accessions. Among the 12 sub populations of 3010 Oryza species, four clusters (East Asian temperate (GJ-tmp), Southeast Asian Subtropical (GJ-sbtrp), South East Asian tropical (GJ-trp) and japx (GJ-japx)) showed a clear 100% presence of the 'G' allele. Whereas, the minor allele predominates in groups containing Aus, Boro and Rayada ecotypes from Bangladesh and India (circum Aus group-cA) and the Basmati and Sadri aromatic varieties (circum Basmati group-cB).

A

B

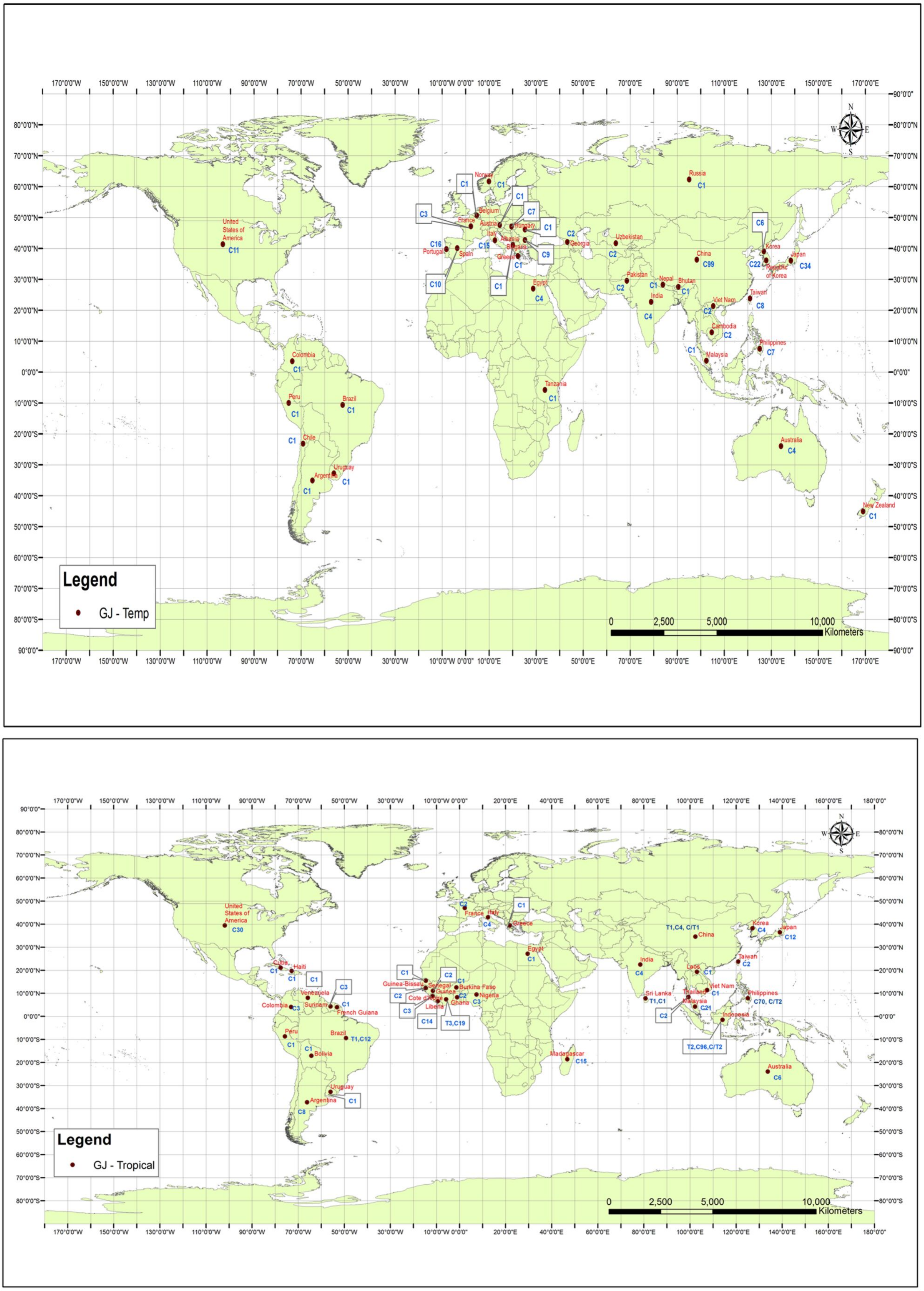

Supplementary Figure 10: Distribution of major allele C(G) of SNP-rs34287911 across the world in 3010 *O. sativa* accessions. A: Country wise distribution count of major or minor alleles ('C' and 'T' respectively) in East Asian temperate (GJ-tmp) accessions and B: Country-wise distribution count of major or minor alleles ('C' and 'T' respectively) South East Asian tropical (GJ-trp) region. Software: Arc Map 10.4.1 version https://tapiquen-sig.jimdofree.com/english-version/free-downloads/world/).

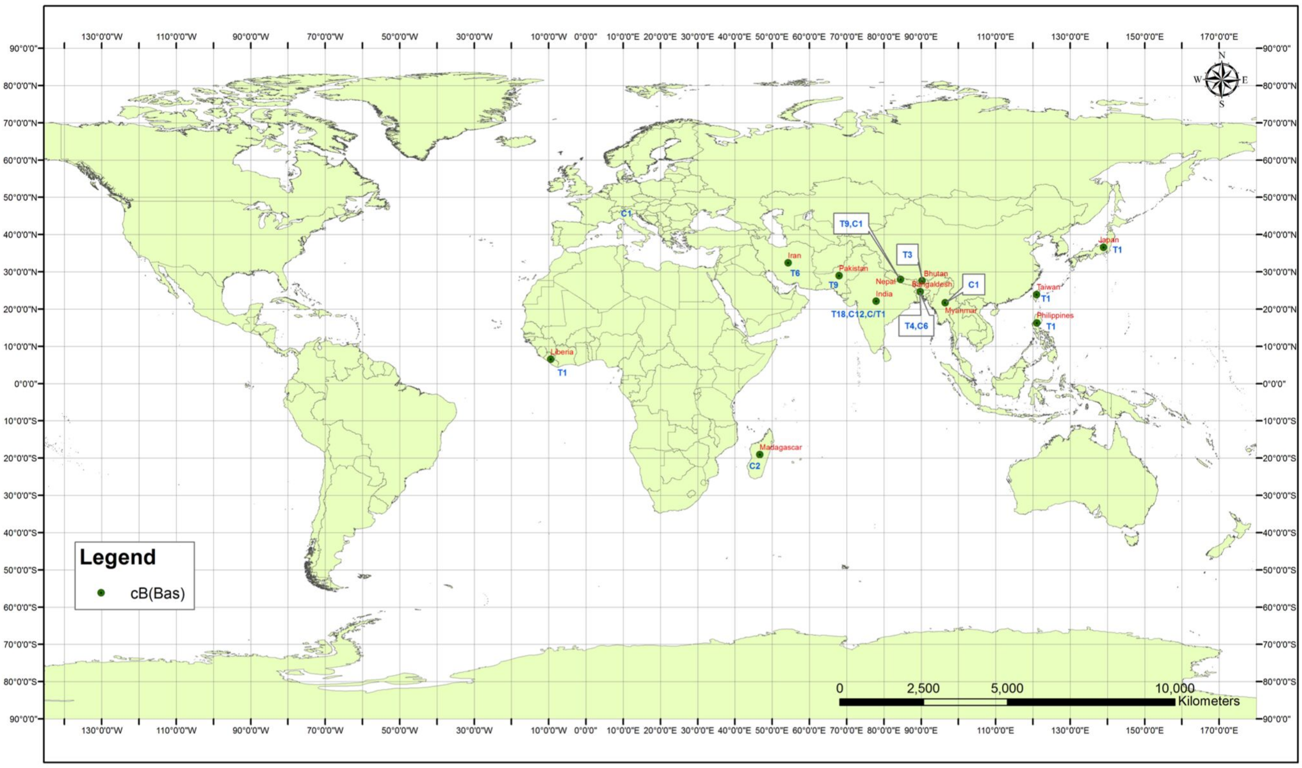

Supplementary Figure 11: Distribution of major allele C(G) of SNP-rs34287911 across the world in 3010 *O. sativa* accessions. A: Country wise distribution count of major or minor alleles ('C' and 'T' respectively) in circum Basmati (cB) accessions Software: Arc Map 10.4.1 version https://tapiquen-sig.jimdofree.com/english-version/free-downloads/world/).

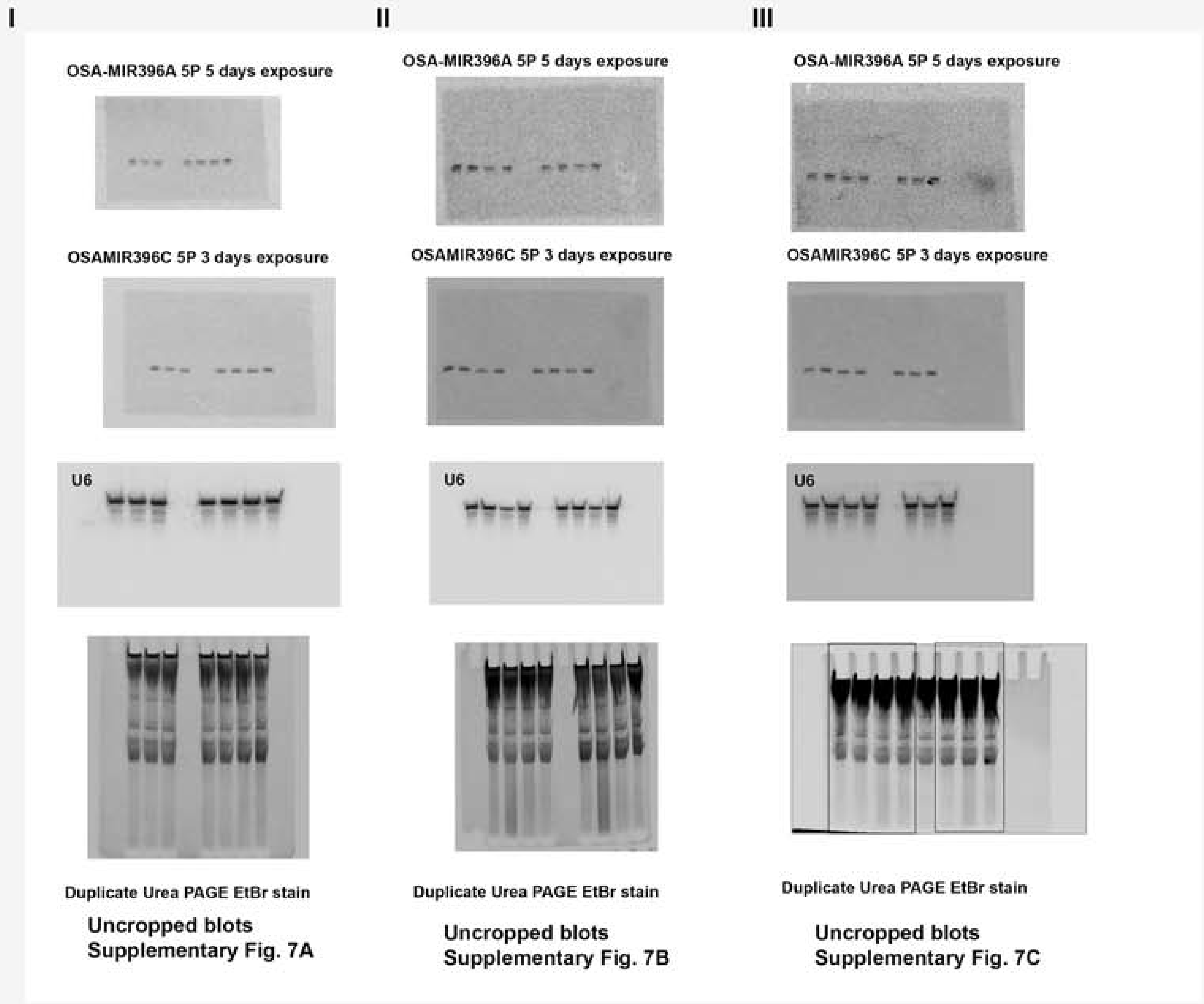

Supplementary Figure 12: Scanned images of Uncropped Northern Blots and ethidium stained gels shown in Supplementary Figure 7A, B and C (top to bottom in I, II and III respectively). For OSA-miR396A and OSA-miR396AC labelling is indicated above Northern blots while for Urea SDS-PAGE gels, labels are indicated below; Blots were probed with U6 as loading control.
